# Messenger RNAs bearing tRNA-like features exemplified by interferon alfa 5 mRNA

**DOI:** 10.1007/s00018-015-1908-0

**Published:** 2015-04-22

**Authors:** Rosa Díaz-Toledano, Jordi Gómez

**Affiliations:** Laboratorio de Arqueología del RNA, Departamento de Bioquímica y Biología Molecular, Instituto de Parasitología y Biomedicina López Neyra (IPBLN-CSIC), Armilla, Granada Spain; Centro de Investigación Biológica en Red de Enfermedades Hepáticas y Digestivas (CIBERehd), Madrid, Spain; Centro de Biología Molecular Severo Ochoa (UAM-CSIC) Cantoblanco, Madrid, Spain

**Keywords:** RNA mimicry, RNase P, IFNA, CAR signal, HCV IRES, L-shaped

## Abstract

The purpose of this work was to ascertain whether liver mRNA species share common structural features with hepatitis C virus (HCV) mRNA that allow them to support the RNase-P (pre-tRNA/processing enzyme) cleavage reaction in vitro. The presence of RNase-P competitive elements in the liver mRNA population was determined by means of biochemical techniques, and a set of sensitive mRNA species were identified through microarray screening. Cleavage specificity and substrate length requirement of around 200 nts, were determined for three mRNA species. One of these cleavage sites was found in interferon-alpha 5 (*IFNA5*) mRNA between specific base positions and with the characteristic RNase-P chemistry of cleavage. It was mapped within a cloverleaf-like structure revealed by a comparative structural analysis based on several direct enzymes and chemical probing methods of three RNA fragments of increasing size, and subsequently contrasted against site-directed mutants. The core region was coincident with the reported signal for the cytoplasmic accumulation region (CAR) in IFNAs. Striking similarities with the tRNA-like element of the antagonist HCV mRNA were found. In general, this study provides a new way of looking at a variety of viral tRNA-like motifs as this type of structural mimicry might be related to specific host mRNA species rather than, or in addition to, tRNA itself.

**Electronic supplementary material:**

The online version of this article (doi:10.1007/s00018-015-1908-0) contains supplementary material, which is available to authorized users.

## Introduction

The ability of viral mRNAs to mimic tRNA was first discovered more than 40 years ago after observing that the 3′-end of the turnip yellow mosaic virus (TYMV) was capable of undergoing covalent linkage with amino acids catalyzed by valyl-tRNA synthetase [1]. This and other plant viral RNAs were subsequently observed to be accessible to a battery of factors involved in other tRNA-related activities [2, 3], including the accessibility of bacterial RNase P [4] reviewed in [5–9]. Nevertheless, in vivo functional mimicry was not complete, since plant viral RNAs were not amino acid donors for protein synthesis but rather participated in virus replication [10, 11].

RNase P specifically cleaves the tRNA precursor (pre-tRNA) to produce its mature 5′-end [12]. It contains an RNA subunit required for activity [13] (with exceptions [14, 15]), and represents an extremely conserved ribozyme activity such that bacteria and human enzymes recognize and correctly cleave each other’s pre-tRNA substrates. It is found in virtually all organisms, with recognition dependent on structural features of the RNA substrate rather than sequence requirements [12, 16]; reviewed in [17–20]. RNase P is an accepted tool for detecting the presence of tRNA-like structures. Indeed RNase P cleavage studies led to the identification of a number of verified tRNA-like domains in non-tRNA molecules [4, 21–32]. In particular, the RNase P from HeLa cells and the ribozyme moiety from the cyanobacterium *Synechocystis* sp. were found to cleave genomic HCV RNA near the AUG start triplet of the internal ribosome entry site (IRES), thus suggesting a similarity to tRNA [33, 34]. The cleavage region was subsequently shown to adopt an L-shaped structure by cryo-electron microscopy of HCV IRES/40S ribosomal subunit complexes [35] and the use of bioinformatic tools [36]. RNase P was also found to cleave the IRESs of the related animal pestiviruses and the cricket paralysis virus [37]. To date, tRNA-like motifs are the only common structural element to have been found in viral IRES [37–40]. It should be clarified that these are in vitro studies and that there is no evidence that RNase P cleavage takes place within the HCV lifecycle [41–43].

Herein we seek parallels between the known HCV mRNA and the liver host mRNA, using two RNase P activities of different origins and compositions i.e. human RNase P and the ribozyme form *Synechocystis* sp. as probing tools. First, we report a set of cellular mRNAs carrying RNase P-sensitive motifs, and then we characterize a motif in specific liver interferon-alpha subtype 5 (*IFNA5*) mRNA. The secondary structure determined adopted a cloverleaf structure. It coincides with the most conserved secondary structure region among all subtypes of interferon-alpha mRNAs and with a large RNA signal that mediates mRNA localization known as the cytoplasmic accumulation region (CAR).

This represents the first tRNA-like structure determined within a human mRNA, thus sharing this property with bacterial and a yeast mRNAs [24, 25, 44]. We also report the subset of key experiments which indicate that both RNase P activities specifically recognize regions of considerable length in two other mRNAs (i.e. H2AFJ and RSP9 mRNAs), thus making the presence of RNase P substrate elements within mRNAs a putatively extended feature throughout human mRNAs, and potential model structures for viruses to mimic.

## Materials and methods

### DNA templates and in vitro transcription

The RNA transcripts used as standard substrates in the RNase P assays were derived from plasmids, and were performed as previously described [33]. The human genes selected in this study, namely *H2AFJ*, *RPS9* and *IFNA5*, were cloned in pGEM3Zf(−) between the EcoR I and Hind III cloning sites. *H2AFJ* and *RPS9* cDNAs were obtained from a human foetal liver cDNA library (Clontech) using nested PCR with the following set of primers: first PCR round for H2AFJ up GTAAAGAGTTTGTAGAGGCA and for RPS9 up CTCTTTCTCAGTGACCGGGT and the common down primer CTGCAGTTTTTTTTTTTTTTTTTT. Second round PCR for H2AFJ up CATGAATTCGCGGCCGTAAAGAGTTTGTAGA and down AGTAAGCTTTCACCAACTTTATTGGCTCC; and for RPS9 up CATGAATTCCTCTTTCTCAGTGAC and down AGTAAGCTTTTTGTAAAGCGCTGA. The* IFNA5* DNA clone (MHS1010-98052299/Clon Id.7939602) was purchased from Open Biosystem. The three plasmids (pGEM3Z-IFNA5, pGEM3Z-H2AFJ and pGEM3Z-RPS9) were digested with Hind III to provide RNA transcripts 700, 658 and 714 nts in length. Shortened DNA templates for each gene were obtained by PCR using the corresponding recombinant pGEM3Z DNA as template and synthetic oligonucleotide as primers. The upstream oligonucleotide contained the T7 promoter sequence linked to the specific sequences. These were: IFNA5 197 EcoRI T7-TCTCTCCTTTCTCCTGCCT and Hind III-TCCACTCCAACCTCCTGCAT; IFNA 215 EcoRI T7-TGAAGGACAGACATGACTT and Hind III-TCATACAGGCTTCCAGGTCAT; and IFNA5 329 EcoRI T7-TCAGCACAAAGGACTCATC and Hind III-TCATACAGGCTTCCAGGTCAT; T7 H2A 415 TAATACGACTCACTATAGGGACCATCGCTCAGGGCGGCGTC; T7 H2A 451 TAATACGACTCACTATAGGGCTGCTGCCCAAGAAGACGGA; H2A 657 (−)CACCAACTTTATTGGCTCCC; H2A 609 (−)CTAGATGTCACCGGCCCTCC; H2A 643 (−)GCTCCCGCCGGGACCCTC; T7 RPS9 8 TAATACGACTCACTATAGGGCAGTGACCGGGTGGTTTGCT; T7 RPS9 27 TAATACGACTCACTATAGGGTTAGGCGCAGACGGGGAA; T7 RPS9 159 TAATACGACTCACTATAGGGTATGGGCTCCGGAACAAACGT; RPS9 236 (−)CAGTTCCCGGGCGGCCTT; RPS9 215 (−)GATCTTGGCCAGGGTAAAT.

The fragments resulting after transcription were *IFNA5* RNA (197–446), (215–427) and (397–427); *H2AFJ* RNA (415–657), (456–657), (456–643) and (456–609) and *RPS9* RNA (8–236), (27–215), and (159–215).

To obtain internally labelled substrates, 5′-end-labelling and 3′-end-labelling reactions, for the cleavage assays, we followed the protocol was described in Ref. [45].

The nucleotide sequences for the transcripts used in the study are obtained from the GeneBank database under accession numbers NM_177925.1 (*H2AFJ*), NM_001013 (*RPS9*) and NM_002169 (*IFNA5*).

### *Synechocystis* sp. RNA ribozyme preparation

Plasmid pT76803 containing *Synechocystis* sp. PCC6803 RNase P RNA [46] was digested with Dra I to provide a transcript of 437 nts. A total of 2 µg of DNA template were then transcribed using the MEGAscript^®^ kit (Ambion) and the RNA purified using MEGAClear™ (Ambion) columns. Its activity was titrated against internally labelled pre-tRNA^Tyr^. Small aliquots were then stored at −80 °C until further use. The presence of cytosines in the 3′ terminal sequence in the substrate are not important for the activity of *Synechocystis* sp. ribozyme as it occurs with M1 RNase P ribozyme from *E.coli* [20, 47, 48].

### Human liver mRNA poly(A) and Poly-r(A)

Human liver mRNA poly(A) (Ambion) was prepared from DNase-treated total RNA purified twice by oligo dT-cellulose chromatography.

### Partial purification of human RNase P

RNase P activity was determined using 30 g of human HeLa cells (Cilbiotech) purified following the procedure described by Bartkiewicz et al. [49], as modified by Nadal et al. [33], thus allowing the presence of significant RNase MRP in our RNase P peak activity to be excluded.

### RNase P cleavage and competitions assays

**Fig. 1:**
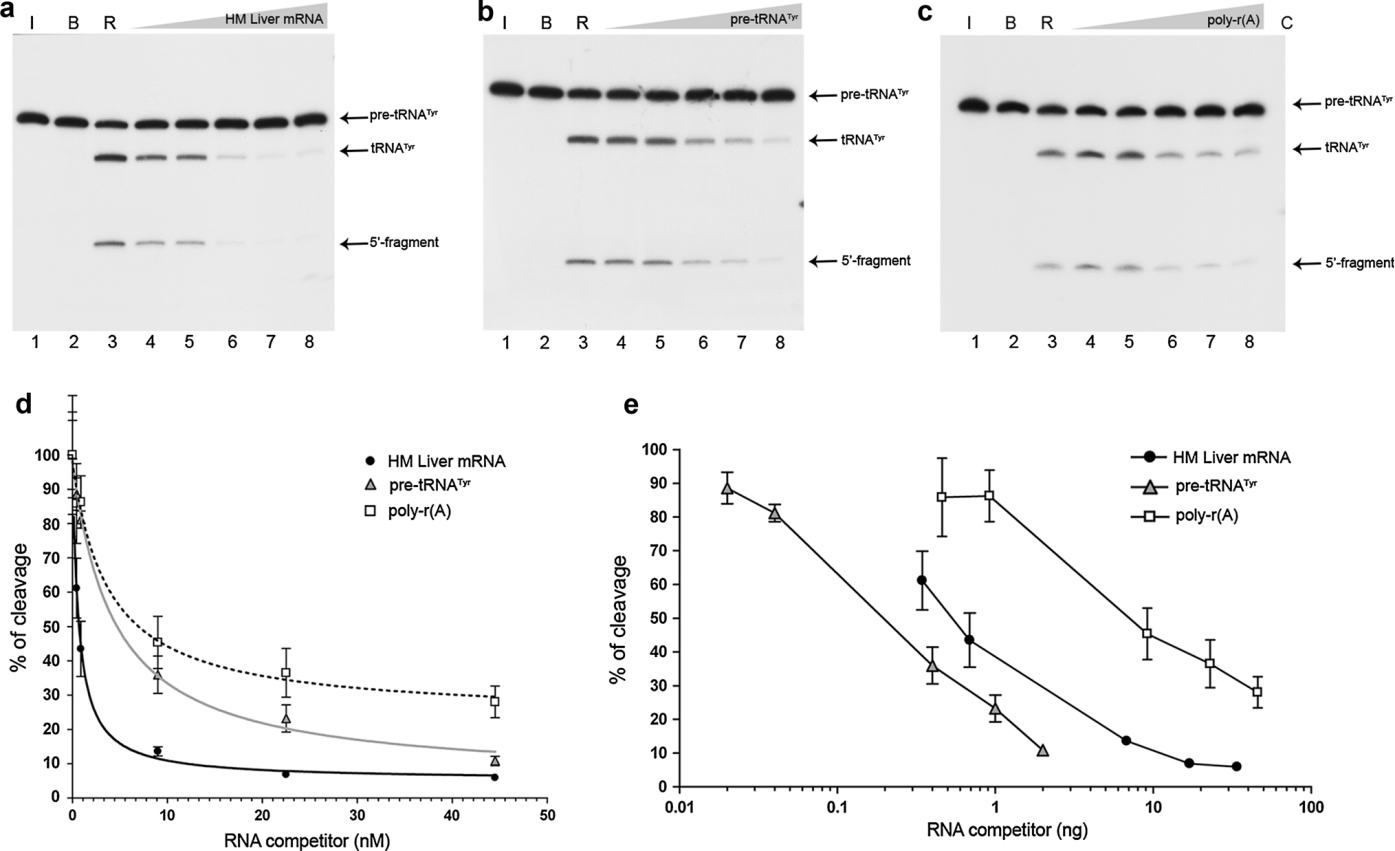
Competitive inhibition of pre-tRNA^Tyr^ processing by human liver mRNA in a standard RNase P reaction. Precursor tRNA^Tyr^ (pre-tRNA^Tyr^: 131 nt) was radiolabelled internally and treated with human RNase P in the absence or presence of increasing RNA competitors. For all panels, *lane 1* shows pre-tRNA incubated on ice (*I*), *lane 2* pre-tRNA in the presence of reaction buffer (*B*), and *lane 3* the control reaction with human RNase P (*R*). *Lanes 4–8* competitive pre-tRNA cleavage with **a** human liver mRNA, **b** cold pre-tRNA^Tyr^ and **c** poly-r(A), at a molar ratio of: 1:0.5, 1:1, 1:10, 1:25, and 1:50, respectively. *Arrows* indicate the main reaction products: tRNA^Tyr^ (88 nt) and a small 5′-fragment (43 nt). **d** Graphical representation of the pre-tRNA^Tyr^ cleavage percentage in the presence of human liver mRNA (*black circles*), pre-tRNA^Tyr^ (*grey triangles*), and poly-r(A) (*unfilled squares*) as competitor RNAs. Data points represent the average of triplicate experiments ± standard deviation. Measurements were normalized taking the control reaction as 100 % of cleavage activity, and adjusted to the equation described in the “Materials and methods” section. The data for poly-r(A) did not fit the equation significantly. **e** As above, except that the cleavage percentage is represented as a function of the competitor RNA weight and the data are not adjusted. Autoradiograms correspond to denaturing polyacrylamide gels at 10 %

Standard reactions were performed as previously described in [33] for human RNase P and [33, 34] for the *Synechocystis* sp. ribozyme, respectively. For competitions using human RNase P, we established an RNase P cleavage inhibition assay in which specificity was monitored in order to determine whether mRNAs from human liver contain structural motifs that competed with tRNA. These inhibition reactions were performed in the linear range for conversion of pre-tRNA^Tyr^ into its products (around 40–50 %; non-saturated reactions Fig. 1). The substrate concentration employed for human RNase P assays (0.9 nM) was lower than the Km (10 nM) reported for the activity purified using the same procedure [50]. Synthetic poly-r(A) (ROCHE) was used as unspecific inhibitor. To establish a molar ratio for the RNA competitors, we assumed an average length of 2250 nts for the mRNA population and 3000 nts for poly-r(A). The inhibition by this polymer of the pre-tRNA^Tyr^ reaction by yeast RNase P is intermediate between those for poly-r(U) and poly-r(G) or poly-r(C) [51].

### RNase P cleavage inhibition: quantification and representation

Quantitative data relating to human RNase P digestion were obtained using an Amersham Biosciences Storm PhosphorImager, and quantified using the Image Quant 5.2 software (Molecular Dynamics). The cleavage percentage was calculated as the ratio of cleavage products/starting material + cleavage products. The values for the different RNA inhibitors were normalized by dividing the rate of product formation in the absence of inhibitor and represented using the GraphPad software (Prism). The data fit to the normalized rate of product formation is given by m1/(1 + (i)/(Ki)) + m3, where m1 is the normalized activity in the absence of inhibitor, (i) the inhibitor concentration, [Ki] the concentration of inhibitor at 50 % inhibition, and m3 the activity extrapolated to saturating inhibitor [52].

### Visualizing mRNA population integrity

The profile of the mRNA population before and after incubation with RNase P activities was evaluated using the automated electrophoresis technique under denaturing conditions with an Experion™ (Bio-Rad).

### Differential screening in microarrays

The Genomics Service at the Centro de Regulación Genómica in Barcelona was responsible for all amplification procedures, labelling the digested RNAs, hybridization and subsequent statistical analysis of data. Human liver mRNA poly(A) samples (500 ng), digested or not with RNase P activities, were amplified by in vitro transcription using the Amino Allyl MessageAmp RNA amplification kit (Ambion). Human Genome 20 K-G4110B (Agilent technologies) was employed for the screening study. The microarrays were hybridized with dye-labelled RNA after amplification of 500 ng of human liver mRNA incubated, or not, with the *Synechocystis* sp. ribozyme at 67.5 nM. Each series consisted of a duplicate hybridisation of dye and dye-swap RNAs. An aliquot of each reaction mixture (control and treated) was incubated in parallel in the presence of two radioactively labelled substrates (HCV RNA 1-570 and pre-tRNA^Tyr^). This control reaction allowed us to follow cleavage by polyacrylamide gel electrophoresis, which showed that the reaction was complete for pre-tRNA^Tyr^, in the expected range of around 10 % for HCV RNA for the ribozyme reaction, and barely detectable for the human RNase P reaction (data not shown).

### RNA end-group analysis and reactions to determine specificity of the cleavage sites

The *IFNA5* (1–700) RNA substrate was labelled at low specific radioactivity to permit an increase of incorporated radioactivity in the subsequent end-labelling reactions, after cleavage with RNase P. The product bands were gel purified and the radioactivity recovered divided into aliquots [45]. Each aliquot was then subjected to specific enzymatic reactions to determine the chemical groups at the 5′- and 3′-ends. For the 5′-end, the RNA was incubated with T4 polynucleotide kinase and [γ-^32^P] ATP with or without phosphatase pre-treatment as described [45]. For the 3′- and 5′-P ends (circular RNA), the product band was incubated with T4 RNA ligase described in Ref. [45]. The reaction products were separated on 4 % denaturing polyacrylamide gels and visualized by autoradiography.

### Structural determination using end-labelled RNAs

#### Enzymes

Single- and double-stranded RNA-specific digestion was carried out under standard conditions, namely, 1000 CPMs of end labelled RNAs incubated: 20 min at 37 °C for RNase T1 (0.001 µg/µL) (Calbiochem), 30 min at 37 °C for RNase V1 (0.0001 U/µL) (Ambion), 1 min 30 s at 37 °C for RNase A (0.0001 ng/µL (Ambion), and 15 min at 37 °C for *E. coli* RNase I (0.01U/µL). RNase concentrations were previously calibrated to give partial cleavage and were run in parallel to: (1) an alkaline degradation sample by heating the RNA during 90 s at 95 °C under conditions which introduced an average of one cleavage per molecule, and (2) a RNase T1 degradation of the RNA transcript under denaturing conditions at 55 °C during 5 min with (0.005 µg/μl). The specificities of the enzymes used for structural analysis are as follows: RNase T1 after unpaired G, pancreatic RNase A after unpaired pyrimidines (C and U), RNase V1 cleaves after any nucleotide either base-paired or single-stranded but stacked [53, 54], and *E. coli* RNase I cleaves are favoured after any single-stranded nucleotide.

#### Chemical reagents

Two specific reagents, namely diethylpyrocarbonate (DEPC), which modifies adenines at the N7 position, and Pb^2+^, which cleaves ssRNA and shows decreased reactivity after guanines, were used.

DEPC modifications were performed under three conditions: native conditions (standard reaction conditions at 10 mM HEPES–KOH pH 7.5, and 100 mM NH_4_OAc), semi-denaturing conditions (10 mM HEPES–KOH pH 7.5, 1 mM EDTA and 100 mM NH_4_OAc) and denaturing conditions. Modification by DEPC was performed according to Peattie and Gilbert [55]. Reaction mixtures of 200 µL containing the appropriate buffer, labelled transcripts (1000 CPM), 10 µL of pure DEPC and 10 µg of tRNA from yeast, were incubated at 30 °C for 10 min. Incubation at 95 °C for 5 min was used in the case of denaturing conditions. After rounds of purification, modified bases were cleaved with aniline [55].

Pb^2+^ reactions were carried out under two different conditions of time and temperature: 30 °C for 20 s, which provided generalized reactivity, and 4 °C for 15 min, in which only a few bases were reactive. Both reactions were performed using 40 mM of Pb(OAc)_2_ pH 7.2 and 1000 CPMs of end labelled transcripts. Products were analysed on denaturing urea gels at two concentrations of polyacryalmide (6 and 10 %), and exceptionally also at 15 % as indicated.

### Structural determination using internally labelled RNA

Internally radiolabelled RNAs were digested for 20 min at 37 °C by addition of 0.01 mg/mL RNase T1 (Calbiochem), an amount that was found to be sufficient to give complete digestion of *IFNA5* RNAs under standard buffer and salt conditions. The reaction products were subjected to 15 % denaturing polyacrylamide gel electrophoresis. Several products considered to be resistant to complete RNase T1 digestion were eluted from this gel and re-digested to completion in a new RNase T1 incubation in 10 mM Tris–HCl pH 7.5 and 1 mM EDTA pH 7.5 for 20 min at 37 °C (secondary digestion). The products of each secondary digestion were re-run in parallel with “sequence” markers in 26 % denaturing acrylamide gels. To obtain the RNA sequence markers, four DNA synthetic oligonucleotides 20 to 25 nts in length sequentially comprising most of the RNA sequence subjected to analysis (positions 329–427) and carrying the T7 promoter were transcribed in vitro in the presence of [α-^32^P]GTP or [α-^32^P]UTP and purified on 20 % denaturing polyacrylamide gels. The DNA oligonucleotides were:

T7-IFNA5 349-328 (−): CAGATGAGTCCTTTGTGCTGACCCTATGAGTCGTATTA; T7-IFN5A 388-364 (−): CAGTGTAGAATTTGTCTAGAAGTGTCCCTATAGTGAGTCGTATTA; IFNA5 399-374 (−): CTGGTAAAGTTCAGTGTAGAATTTGTCCCTATAGTGAGTCGTATTA; T7-IFN5A 400-418 (−): CTTCCAGGTCATTCAGCTGCCCTATAGTGAGTCGTATTA.

The gel-purified transcripts were totally digested with RNase T1, and their products identified on the basis of their relative migration pattern and differential labelling intensity depending on whether they were “G” or “U” labelled.

In the 26 % polyacrylamide electrophoretic run, the re-digestion lane for the protected RNA species gives a more or less complex mixture of products that can be identified by comparison of their mobility with the corresponding digestion product of the marker.

### Sequencing of RNase P cleavage products

#### Direct RNA sequencing

End-labelled RNase P cleavage products were run in denaturing 6 % polyacrylamide gels in parallel to alkaline degradation and RNase T1 ladder of the same RNA transcripts prepared as described in the previous paragraphs.

#### Indirect sequencing

Indirect DNA sequencing was employed to sequence *IFNA5* (1–700) RNA cleavage products of human RNase P. Addition of a track of poly(A) or poly(U) to the new 3′-end products of RNase P, or cyclising those RNA products that present a new 5′-end, allows us to use a complementary oligonucleotide to prime cDNA synthesis, followed by RT-PCR, cloning using pGEM-T^®^ Easy (Promega), and Sanger sequencing analysis.

## Results

### Analysis of the presence of human RNase P cleavage sites in liver mRNAs by enzymatic competition

We tested the ability of increasing amounts of human liver mRNA to compete for RNase P cleavage of the natural pre-tRNA^Tyr^ substrate internally labelled with [α-^32^P]GTP (Fig. 1a). The results of this experiment were compared with those for an assay in which cold pre-tRNA substrate was used to compete with its labelled form (Fig. 1b). Synthetic poly-r(A), which is not a substrate for RNase P [56], was used to evaluate the non-specific competition effect by increasing the amount of RNA in the reaction mixtures (Fig. 1c).

The amount of cold pre-tRNA^Tyr^ required for half-inhibition of cleavage was 4.36 ± 1.6 nM, whereas liver mRNA was a stronger inhibitor (half-inhibition at 0.63 ± 0.17 nM; Fig. 1d). In contrast, when the inhibitory activity was expressed in terms of mass, 4.8 times more mRNA than cold pre-tRNA^Tyr^ was needed to reach half-inhibition (Fig. 1e). Synthetic poly-r(A) was the least competitive in both cases. Considered together, these results indicate that the observed inhibition of pre-tRNA^Tyr^ processing by human liver mRNA is at least partially specific and also point to a molecular similarity between the internal motifs embedded within mRNA and pre-tRNA. Nevertheless, the cleavage-product distribution after human RNase P treatment of mRNA showed no changes with respect to the control when visualized by automated denaturing electrophoresis (Fig. S1). This indicated that liver mRNA species either did not contain built-in significant RNase P-susceptible tRNA cleavable motifs or, if present, they were not cleaved at a sufficiently high level to be detected by automated electrophoresis.

### Selection of a subset of three candidate mRNAs and direct evaluation of RNase P processing of the transcripts in vitro

We subsequently tried to identify the individual mRNA species within the bulk liver mRNA that carried RNase P cleavage sites in a microarray screening. Details are described in Fig. S2. The in vitro transcribed and purified *Synechocystis* sp. RNase P ribozyme was employed because of its purity with respect to human RNase P extract, and because similar substrate specificity between human RNase P and *Synechocystis* RNase P has been found previously in HCV [34, 57, 41] both are independent of –CCA sequence in the substrate. A list of these genes and their characteristics can be found in Supplementary Table S1. We selected three short mRNAs, ~700 nts in length, to allow analysis of the full length of the cleavage products through gel electrophoresis, and to represent evolutionarily separated activities. These mRNAs were: ribosomal *RPS9*, histone *H2AFJ* and a member of the interferon alfa subtype.

**Fig. 2:**
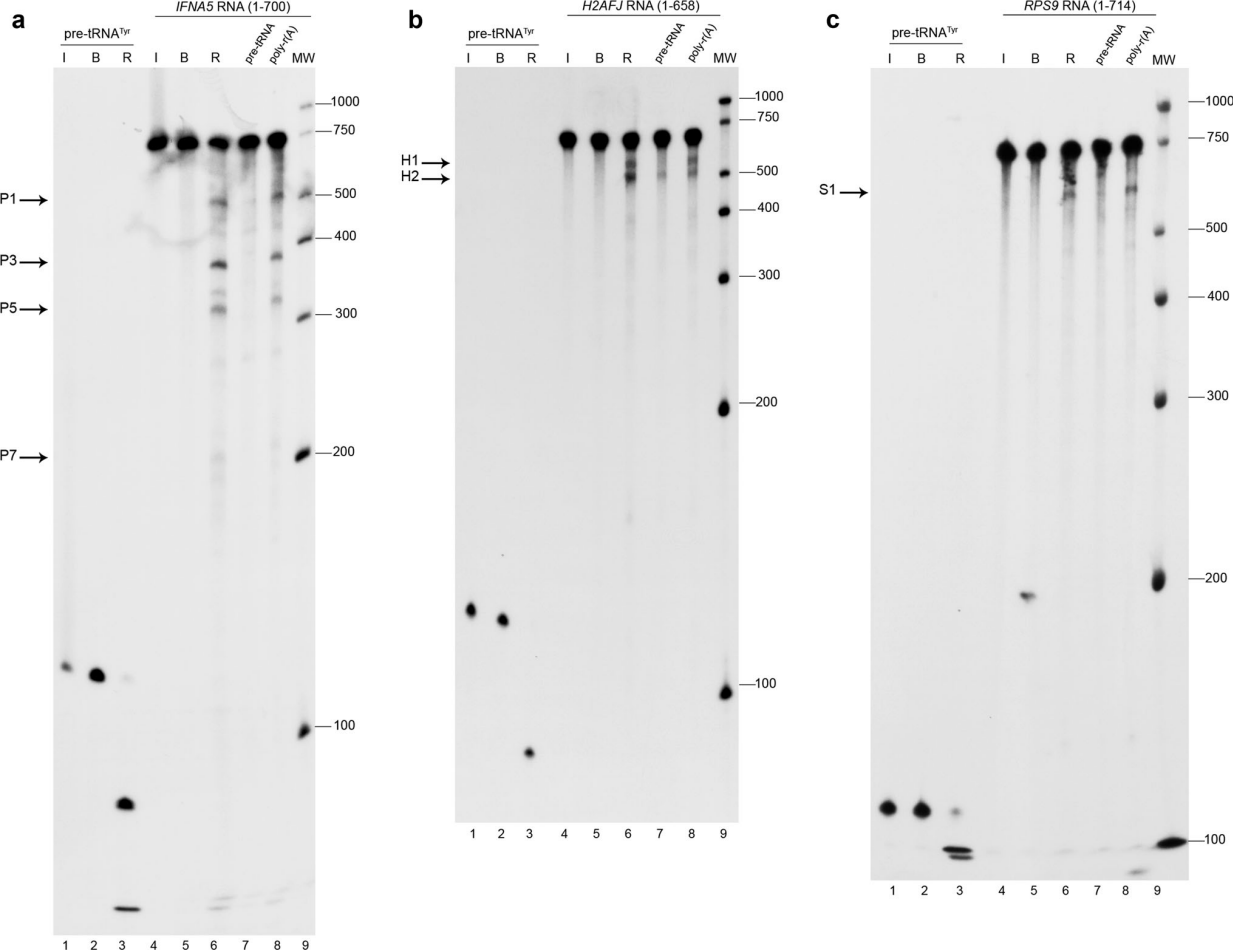
Specific cleavage of *IFNA5*, *H2AFJ* and *RPS9* RNAs by human RNase P. All transcripts were internally radiolabelled during in vitro transcription. The RNA substrate for *lanes 1–3* for all panels is the pre-tRNA^Tyr^ of *E. coli*. *Lanes 4–9* correspond to: *IFNA5 RNA* (1–700) for **a**, *H2AFJ* RNA (1–658) for **b** and *RPS9* RNA (1–714) for **c**. In all autoradiograms: *lanes 1* and *4* correspond to RNAs incubated on ice (*I*); *lanes 2* and *5* in the presence of reaction buffer (*B*); *lanes 3* and *6* reaction with human RNase P (*R*). *Lane 7* human RNase P reaction incubated with a tenfold molar excess of cold pre-tRNA^Tyr^. *Lane 8* incubated with 400 pg of a non-specific competitor poly-r(A). This corresponds to the amount of RNA by weight used in the tenfold pre-tRNA^Tyr^ treatment in *lane 7*. *Lane 9* molecular weight markers. The main digestion products are indicated by *arrows* on the *left* of each panel. The autoradiograms correspond to denaturing polyacrylamide gels at 4 %

The results obtained upon digesting each of the in vitro transcript RNAs with human RNase P are presented in Fig. 2 and after digesting with *Synechocystis* sp. ribozyme in Fig. S3; cleavage reactions were run in parallel with specificity test reactions or in dose–response assays, respectively. In light of the electrophoretic profiles and intensity of the cleavage band products for human RNase P cleavage, we could tentatively assign two primary RNase P cleavage sites for *IFNA5* mRNA, at least one for *H2AFJ* mRNA (see following section) and one for *RPS9* mRNA. The cleavage percentage ranged from approximately 4–15 %. The percentage of cleavage products for *IFNA5* RNA from three independent experiments was 10.1 ± 2.9 % for P1 + P7 and 14.4 ± 4.8 % for P3 + P5.

### Cleavage characterisation: positioning and shortening substrates

**Fig. 3:**
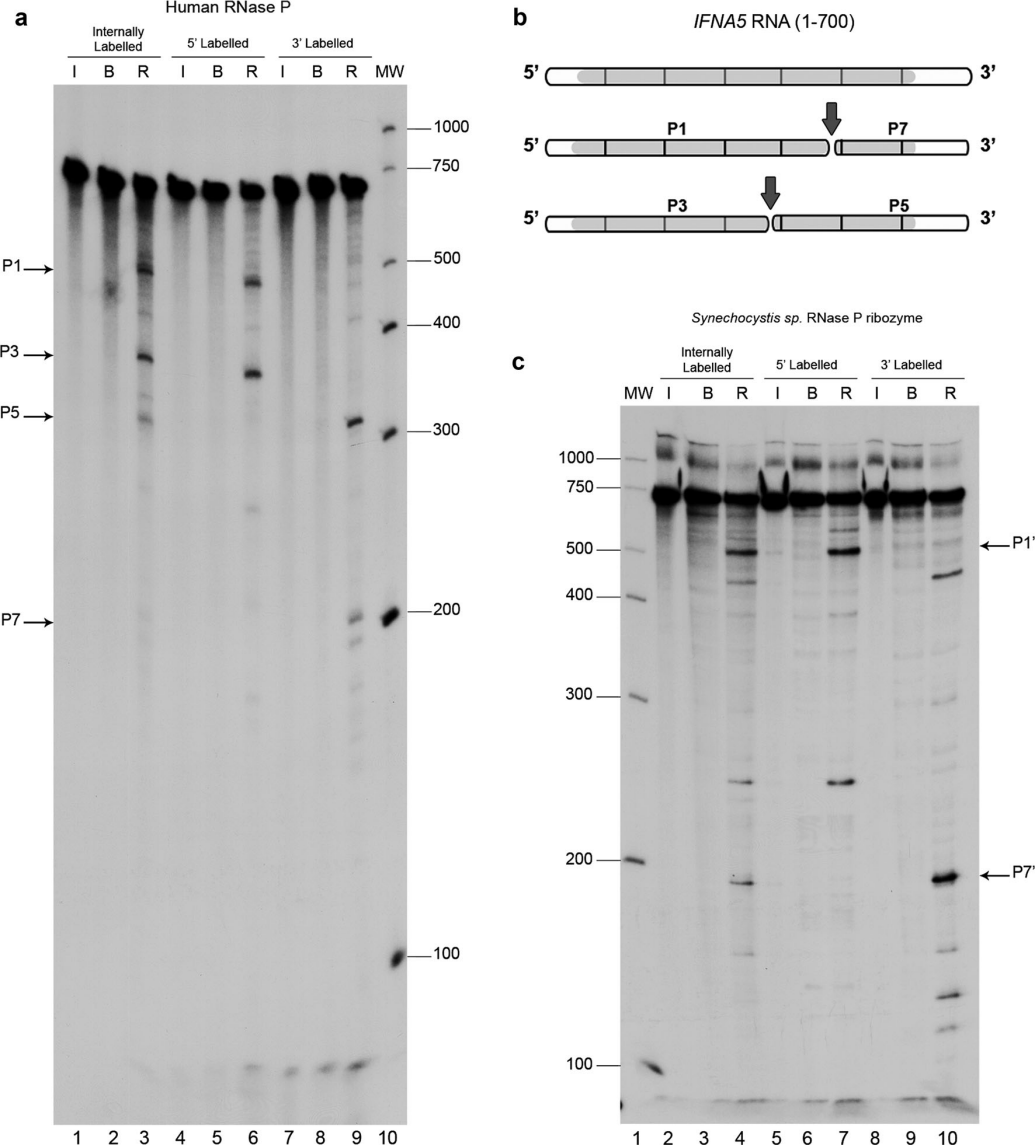
Mapping the cleavage sites in mRNA *IFNA5*. **a** Autoradiogram of human RNase P cleavage of internally radiolabelled (*lanes 1–3*), 5′- (*lanes 4–6*) and 3′ end-labelled (*lanes 7–9*) *IFNA5* transcripts, respectively. *Lanes 1, 4* and *7* RNA incubated on ice (*I*); *lanes 2, 5* and *8* RNA incubated in reaction buffer (*B*); *lanes 3, 6* and *9* reactions with human RNase P (*R*). *Lane 10* is a molecular weight ladder. *Arrows* indicate the major digestion products, designated as P1, P3, P5 and P7. **b** Linear diagram of the *IFNA5* substrate transcript (1–700 nt) segmented every 100 nts. *Grey areas* indicate the protein-coding region and *white areas* the untranslated flanking regions. The final cleavage products (P1–P7) and (P3–P5) deduced from the observed bands on a 4 % polyacrylamide electrophoresis gel are represented below. **c** *Synechocystis* sp. RNase P ribozyme digestion of internally radiolabelled (*lanes 2–4*), 5′- (*lanes 5–7*) and 3′ end-labelled (*lanes 8–10*) *IFNA5* transcripts. *Lane 1* molecular weight ladder. *Lanes 2, 5* and *8* RNA incubated on ice. *Lanes 3, 6* and *9* RNA incubated in reaction buffer. *Lanes 4, 7* and *10* reactions with *Synechocystis* sp. ribozyme. Product bands migrating at similar positions as human RNase P product are marked on the *right-hand side*

**Fig. 4:**
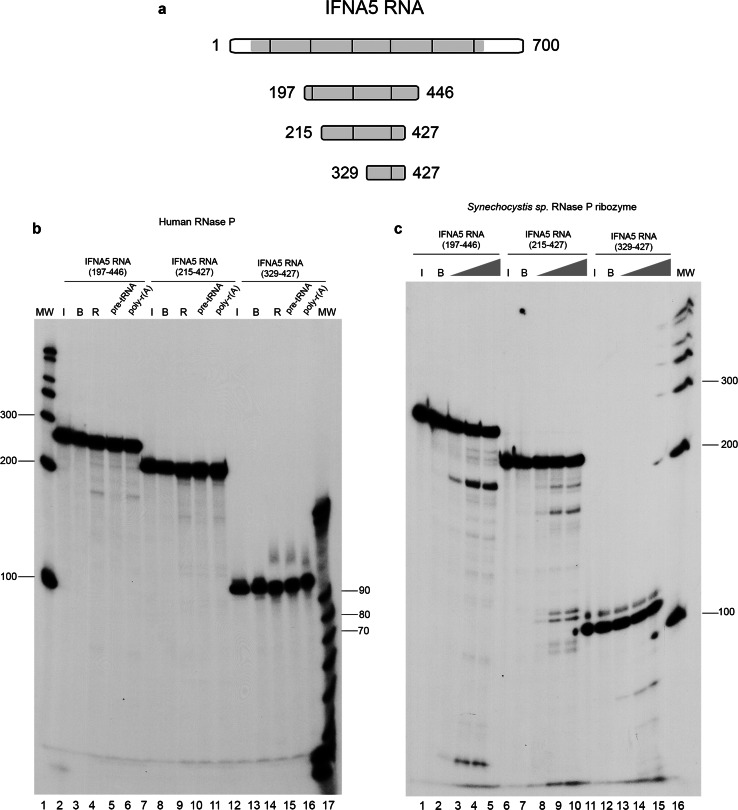
Determination of RNase P minimal substrate and cleavage specificity in the tRNA-mimic region 1. To delimit the human RNase P minimal substrates in *IFNA5* mRNA, shortened DNA templates were obtained by PCR, T7 in vitro transcribed in the presence [α-^32^P]GTP and subjected to cleavage. **a** Schematic representation of shortened *IFNA5* RNA transcripts (197–446), (215–427) and (329–427). **b** Human RNase P activity test under standard conditions. The three fragments were incubated on ice (*lanes 2, 7* and *12*), in reaction buffer (*lanes 3, 8* and *13*), or in the presence of human RNase P (*lanes 4, 9* and *14*). Unlabelled pre-tRNA^Tyr^ (10× molar excess) or poly-r(A) (equivalent quantity by weight) was added to the reaction in *lanes 5, 10* and *15* and *6, 11 * and *16*, respectively. *Lanes 1* and *17* are the century and decade molecular markers, respectively. **c** Cyanobacterial ribozyme dose–response essay. The three fragments were incubated on ice (*lanes 1, 6 * and *11*), in the presence of buffer (*lanes 2, 7* and *12*) or with bacterial ribozyme at 67.5 nM (*lanes 3, 8* and *13*), 337.5 nM (*4, 9 *and *14*) and 675 nM (*lanes 5, 10* and *15*), respectively. *Lane 16* contains the century molecular markers

Differential RNA labelling (5′-end, 3′-end and internal label) afforded direct mapping of the cleavage sites within the three mRNAs (Fig. 3a, Fig.  S4A and B). Results are represented schematically in Fig. 3b and Fig. S4C and D. The shortest RNA fragments tested and which were observed to be cleaved by human RNase P with the same specificity than the full length mRNAs, were *IFNA5* (215–427) RNA (Fig. 4), *H2AFJ* (456-643) RNA (Fig. S5A) and *RPS9* (27–215) RNA (Fig. S5B and C). On average, minimal substrates were 195 nts long. Specificity data were obtained by competitive analysis, as described previously. In the case of *IFNA5* RNA, additional dose–response behaviour was observed for the cyanobacteria ribozyme (Fig. 4d).

### Determination of the *IFNA5 mRNA* RNase P cleavages sites and chemical endgroup analysis

#### Endgroups

The characteristic RNase P endgroups were found in the newly generated termini (i.e. 5′-P for P5 and P7 products and 3′-OH for P1 and P3 products) using three different assays that included phosphatase, kinase and ligase treatment, as described in the “Materials and methods” section and Supplementary Fig. 6.

#### Sequencing

An understanding of the cleavage polarity and end-group chemistry for each product RNA allowed us to design a strategy to generate a template for the cDNA synthesis primer. Bands P5 and P7 were circularised while enzymatic addition of poly(A) and poly(U) were employed for bands P1 and P3. Supplementary Fig. 7A summarizes the results obtained for RT-PCR cloned products P1, P3, P5 and P7. Cleavage was found to be distributed over several neighbouring sites for all products rather than being pinpointed as a specific single site. The most frequently identified cleavage site positions were A_376↓_A_377_ for P3, A_377↓_A_378_ for P5, G_488↓_A_489_ for P7 and A_491↓_A_492_ for P1. Direct RNA sequencing of the proximal cleavage site (between band products P5 and P7) indicated that cleavage occurred approximately after base A_377_ (Fig. S8), which coincided with cleavage at A_377_↓A_378_ found for the most represented product end-group of P5 in cDNA sequencing.

### Structural analysis of the *IFNA5* RNA in the proximal RNase P cleavage site

**Fig. 5:**
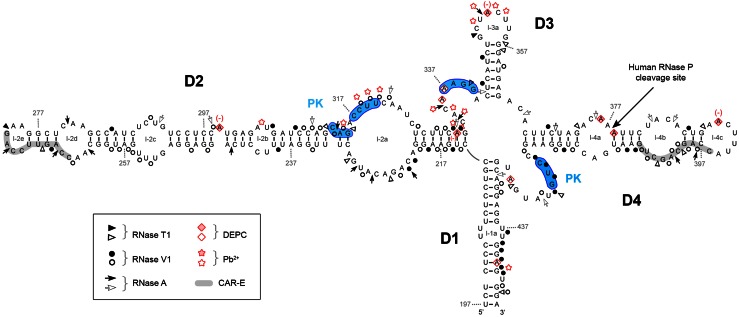
Summary of the enzymatic and chemical probing results for *IFNA5* RNA (197–446). Mapping data are depicted upon common predicted secondary structure of different species using Centroid program http://www.ncrna.org/centroidfold/. Enzymatic cuts are showed as *arrow heads* (RNase T1), *circles* (RNase V1) and *arrows* (RNase A). The bases modified by DEPC are indicated by *rhomboids* and those cleaved by Pb^2+^ by *stars*. Strong cleavages are indicated by *filled symbols* and moderate ones by *unfilled symbols*. (+) and (−) enhanced or protected cleavage in MgCl_2_. *Numbers* indicate nucleotide positions. *D* domain, *l* loop, *PK* possible pseudoknot. In *blue*, sequences participating in a predicted pseudoknot. Highlighted in *gray* are CAR-E sequences

The structure prediction model for interpreting the experimental analysis was the common secondary structure observed for *IFNA5* (197–446) in several mammals species, obtained using the Centroid program (Fig. 5). This model comprises a four-branched structure; domains are connected by a central loop and are designated as domain 1 to domain 4 (D1–D4) in clockwise direction. Helices and loops within each domain will be designated as stem (s) or loop (l) followed by letters. Three RNA fragments were analysed by chemical and enzyme probing experiments: *IFNA5* RNA (197–446), which contained domains D1 to D4 and was cleaved by RNase P; *IFNA5* RNA (215–427), which contained domains D2 to D4 and was also cleaved by RNase P, although at a slower rate; and *IFNA5* RNA (397–427), which contained D3 and D4, but was not cleaved by RNase P even though it contains the cleavage site. This three fragment mapping strategy, with each fragment having successively one less domain, was previously used for TMV plant tRNA-like characterisation [58].

### RNase probing reactions of 5′- and 3′-end-labelled transcripts

#### *IFNA5* RNA (197–446)

**Fig. 6:**
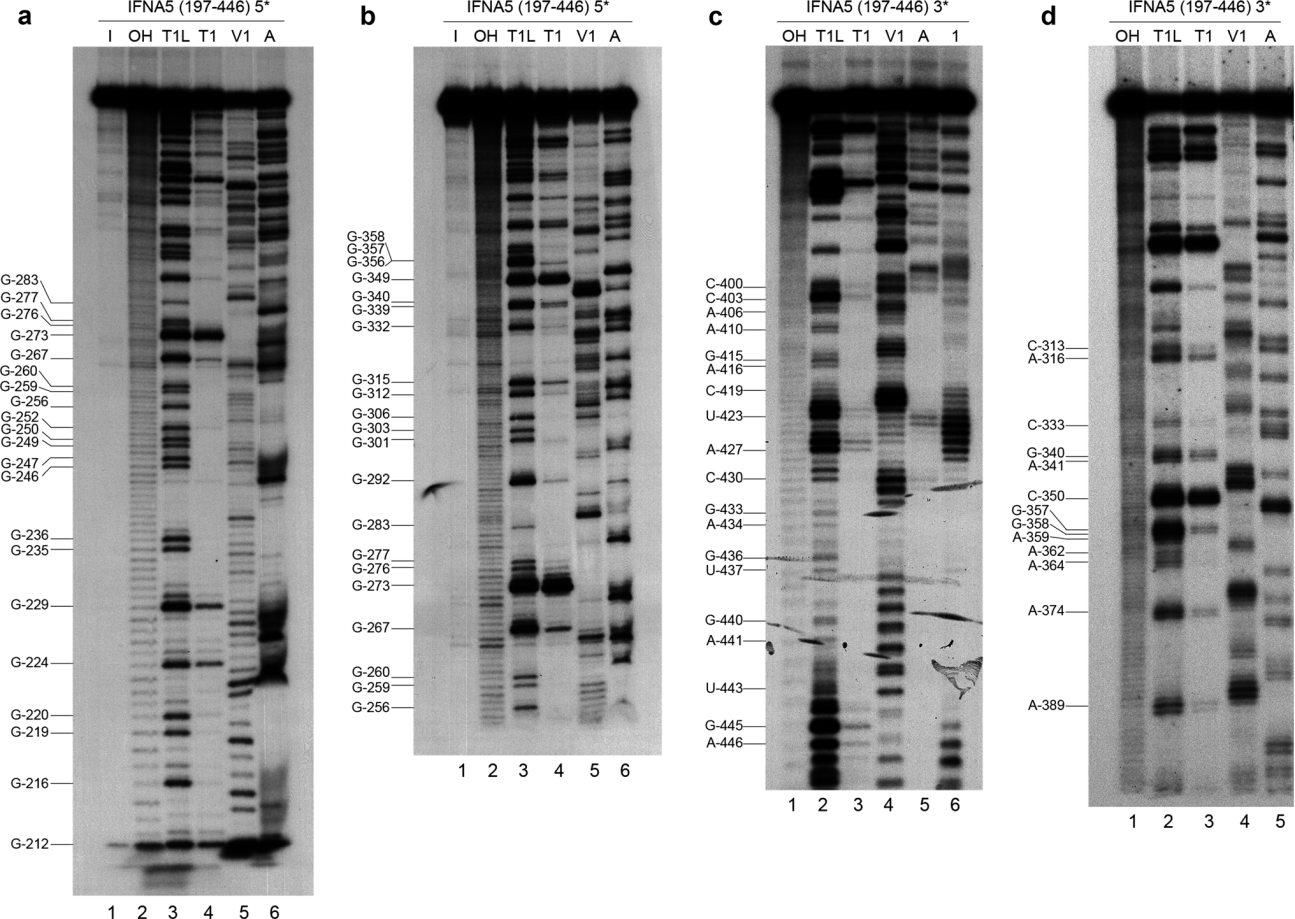
Enzymatic probing of the secondary structure of *IFNA5* RNA (197–446). **a**, **b** 5′-[^32^P] end-labelled RNA. **b**, **c** 3′-[^32^P] end-labelled RNA. For **a** and **b** *lane 1* is the RNA maintained on ice (*I*); *lane 2* alkaline hydrolysis reaction (*OH*); *lane 3* RNase T1 reaction under denaturing conditions (*T1L*); *lane 4* RNase T1 (*T1*), *lane 5* RNase V1 (*V1*) and *lane 6* RNase A (*A*) under standard conditions, respectively. For **c** and **d** products digested with alkali (*OH*), RNase T1 under denaturing conditions (*T1L*), RNase T1 (*T1*), RNase V1 (*V1*), RNase A (*A*) and *E. coli* RNase 1 (*1*) under standard conditions were analysed in *lanes 1* to *6*, respectively. Denaturing gels were at 10 (**a**), 6 (**b** and **d**) and 15 % (**c**) polyacrylamide. The *numbers* on the *right* indicate the point of digestion cleaved by RNase T1 under denaturing conditions (*T1L*), as identified with the help of the OH sequence ladders run on the *left*

Figure 6 show images of the denaturing polyacrylamide gels of the sequential base-sensitivity for RNases T1, V1 and A for the *IFNA5* RNA (197–446) fragment. In the case of the 3′-end-labelled transcript, *E.coli* RNase I probing was also performed. Cleavages were mostly consistent with the predicted structures of the individual stems of D1 to D4, and also for the apical loops D2 and D3, although there were discrepancies in either the junction of these domains, in the apical loop of D4 or in the internal bulges (Fig. 5). In a clockwise direction, these discrepancies were: (1) in the two opposite sequences forming loop 2a: the first sequence motif A_221_–A_230_ contained two strong and two moderate RNase V1 cleavages, and the second motif A_316_–C_325_ contained a set of four mild RNase V1 cleavages in its central portion, thus indicating that this region is more complex than a single-stranded RNA bulged motif; (2) in loop 4b, which is comprised of mild RNase V1 cleavages and a coincident RNase T1 and RNase V1 mild cleavage at G_402_; (3) the apical loop sequence 4c (positions A_389_–C_396_) lacked the expected cleavages by single-stranded RNases; and (4) the predicted single-stranded RNA junction region between D1 and D4. This central region contained a stretch of strong RNase V1 cleavages (C_419_CUG_422_), thereby ruling out its single-stranded RNA nature.

#### Comparative analysis of the three RNA fragments

Structural data from the three *IFNA5* RNA fragments (197–446) (Fig. 6), (215–427) (Fig. S9) and (329–427) (Fig. S10) were compared to obtain information about possible interactions between the D1 to D4 domains that could explain the detected incompatibilities.

**Fig. 7:**
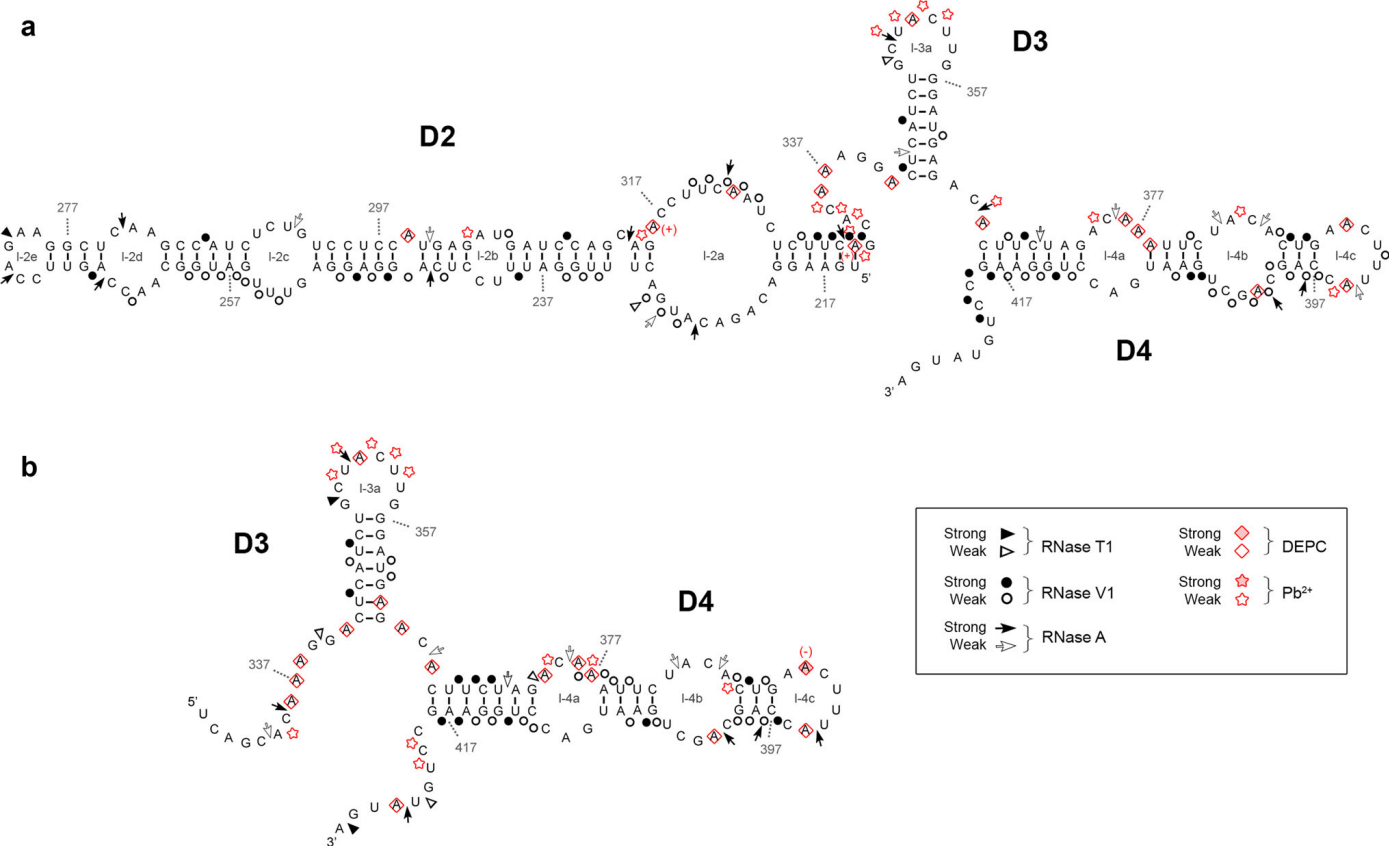
Summary of the enzymatic and chemical probing results for *IFNA5* RNAs (215–427) (**a**) and (329–427) (**b**). Idem as figure

The main difference between *IFNA5* (197–446) RNA and *IFNA5* (215–427) RNA (Figs. 5, 7a) was the disappearance of two strong RNase A cleavages; C_333_ and C_335_ in the junction between D2 and D3 for the case of *IFNA5* (215–427) RNA and its mild RNase V1 cleavages in C_313_AG_315_. The remaining differences were minimal, supporting the idea that the fragments are structurally equivalent.

The most important changes occurred between *IFNA5* RNAs (197–446) and (215–427) (Figs. 5, 7a) on the one hand and (329–427) (Fig. 7b) on the other. The most remarkable of these changes was the disappearance of strong RNase V1 cleavages after bases C_420_UGU_423_ and, to a lesser extent, loss of slight RNase V1 cleavages in G_402_CU_404_ in *IFNA5* (329–427) RNA. As these changes were not compensated by any other coherent changes that might presuppose an internal refolding of shorter *IFNA5* (329–427) RNA when alone, and as these two regions were conflictive in the larger fragment *IFNA5* (197–446) RNA, it can be inferred that these sequences interact with other sequences in domain 2 (common to *IFNA5* (197–446) and (215–427) RNAs), pointing to a pseudoknot structure.

When comparing the size of the three RNA fragments, a particular alteration occurred in predicted stem 4a (C_367_UU_369_ and opposite strand A_416_AG_418_). As domains D1 and D2 are successively deleted, more RNase V1 cleavages were observed, thus indicating that this region is highly inaccessible for the enzyme in the complete fragment.

### Chemical structure analysis

**Fig. 8:**
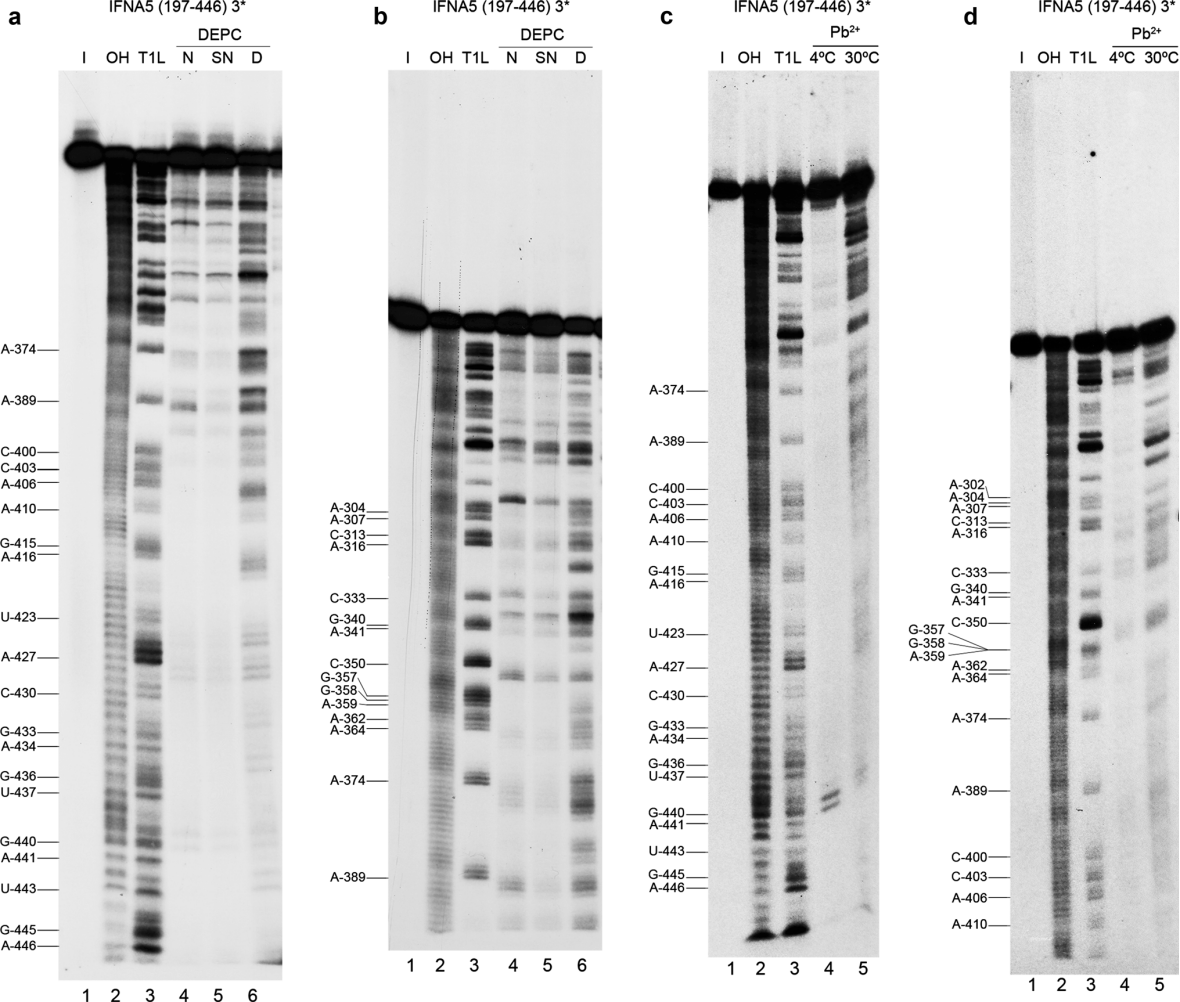
Chemical probing of 3′ end-labelled *IFNA5* RNA (197–446). **a**, **b** DEPC probing. *Lane 1* is the RNA maintained on ice (*I*). *Lanes 2* and *3* are the products from digestion with alkali (*OH*) and RNase T1 under denaturing conditions (*T1L*), respectively. Products of aniline-treated RNAs previously modified with DEPC under native conditions (*lane 4*), semi-denaturing conditions (*lane 5*) and denaturing conditions (*lane 6*). **c**, **d** Pb^2+^ probing. *Lane 1* is the RNA maintained on ice (*I*). *Lane 2* treatment with alkali (*OH*); *lane 3* treatment with RNase T1 under denaturing conditions (*T1L*); *lane 4* treatment with Pb^2+^ at 4ºC for 15 min and *lane 5* treatment with Pb^2+^ at 30ºC for 20 s. Denaturing gels were at 10 % (**a**, **c**) or 6 % polyacrylamide (**b**, **d**)

We performed reactions using 3′-end-labelled RNAs, which allowed us to partially map *IFNA5* RNAs (197-446) (Fig. 8) and (215–427) (Figs. S11 and S12, panels A and B) and completely map *IFNA5* RNA (329–427) (Fig. S11 and S12, panels C and D).

#### *IFNA5* RNA (197–446)

Under restricted probing conditions, Pb^+^ reactivity was concentrated in the central part of internal loop 2a, at the junction between D2 and D3 and in the apical loop of D3 (loop 3a). There was no Pb^2+^-mediated cleavage at all in domain D4, even at its apical loop, despite unpaired sites being predicted (Fig. 5).

Under native conditions, reactivity to DEPC was found at bulged A_299_ in domain 2, at the D2–D3 junction region, in apical loop 3a, internal loop 4a, apical loop 4c and at the D4–D1 junction.

It is remarkable that, according to the base-paired prediction and RNase V1 sensitivity, most basal stem residues in domain D1, and all those in the stem of D3, were protected against modification by DEPC and Pb^2+^. This is indicative of the high stability of these dsRNA regions. The lack of reactivity of DEPC in internal loop 4b, which also does not react with Pb^2+^, reflects a higher degree of organisation.

There is no effect on DEPC reactivity in the absence of Mg^2+^ at the junction regions of the main helices. In contrast, bulged A_299_ and apical loops 3a and 4c at A_352_ and A_390_, respectively, showed reduced reactivity in the absence of Mg^2+^ ions.

#### Comparative analysis

Chemical probing of the three consecutively shortened RNA fragments shown in Figs. 5 and 7 confirmed that they share similar non-reactivity in the proposed stems and reactivity in apical loop 3a, thereby supporting the overall pattern of sensitivity to ribonucleases. The comparative analysis also highlighted several reactivity changes amongst different sized RNAs. Most of these changes involved acquisition of DEPC reactivity on the shorter *IFNA5* RNAs (215–427) and (329–427), and some of them coincided. Two remarkable changes observed when comparing the smaller RNA fragment *IFNA5* (215–427) with *IFNA5* (197–446) were the loss of three Pb^2+^ cuts in the middle of internal loop 2a C_318_UU_320_ and the acquisition of a DEPC reactive site at the flanking A_322_. Another important difference was the larger change in reactivity of *IFNA5* (329–427) RNA at junction D1–D4 in comparison to *IFNA5* (197–446) and *IFNA5* (215–427) RNAs, which acquired two successive Pb^2+^ cuts at sequence C_419_C_420_ and a new DEPC reaction at the neighbouring site A_424_. One prominent Pb^2+^ site specific cleavage [59] was found at base C_439_, at 4 °C, but not at 37 °C, indicating that the specific structure that supported it was not stable.

#### Mg^2+^ participation

Positions observed to have differential reactivity to DEPC in the presence of Mg^2+^ were: A_316_ and A_331_. The first is found between the two contiguous sequences participating in the pseudoknot (G_312_CAG_315_) and (C_317_CUU_320_) (see below). The second corresponds to the nearest neighbour of G_332_ and is located at the four-way junction, thereby suggesting that Mg^2+^ participates in both the four-way junction motifs and in the pseudoknots.

### RNase protection assay using internally labelled RNAs

**Fig. 9:**
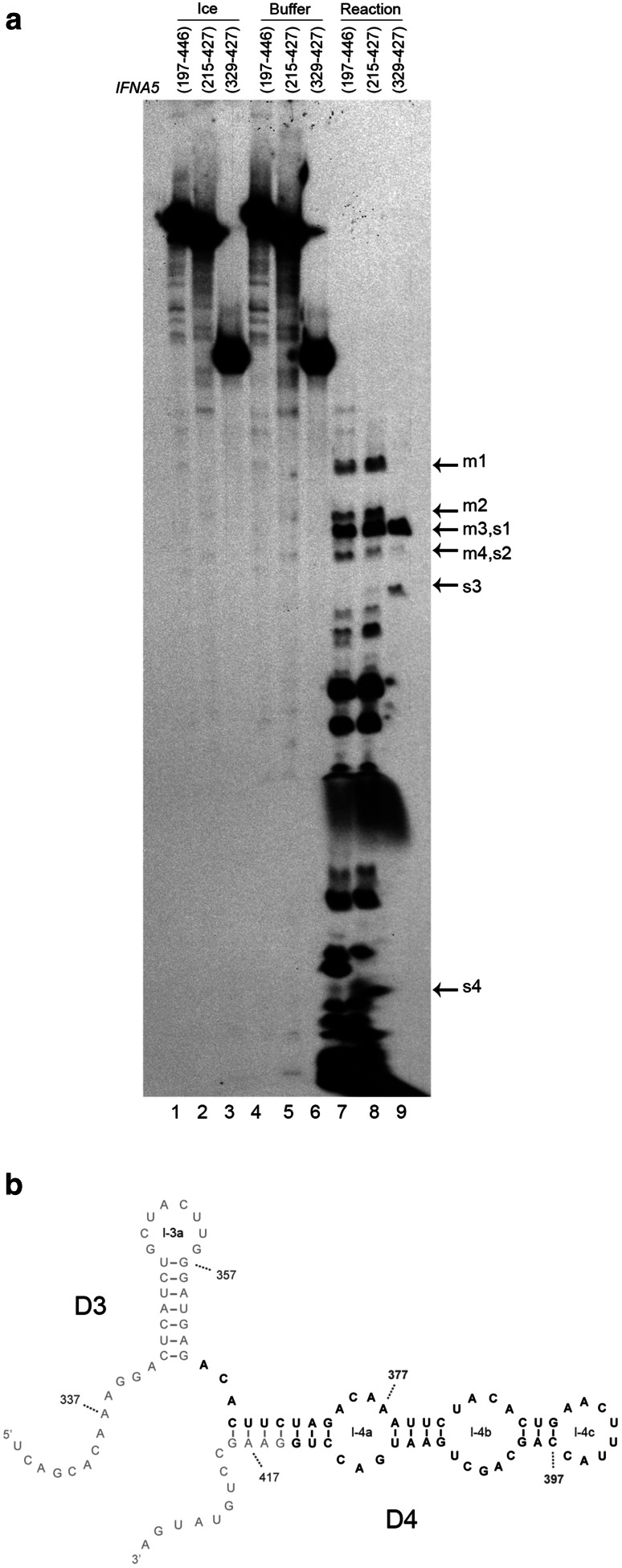
Regions of *IFNA5 *RNA subdomains protected from RNase T1 digestion under native conditions. **a** Protection of *IFNA5* RNAs from RNase T1 digestion assay. Internally labelled *IFNA5* RNAs (197–446), (215–427) and (329–427) maintained on ice (*lanes 1–3*), incubated with digestion buffer (*lanes 4–6*), or in the presence of RNase T1 at saturated concentration (*lanes 7–9*). Prominent product species like those indicated by *arrows* were eluted for further study (supplementary Fig. 14). Denaturing gel was at 15 % polyacrylamide. **b** Region protected from RNAse T1 digestion bases written in *bold* are more protected from digestion with RNase T1 than the ones in standard style. *Numbers* indicate the nucleotide positions

Specific RNA:RNA contacts may help to protect the sequences involved in such contact areas from complete digestion by single-stranded ribonucleases [60, 61]. Thus, another approach to structural comparison involves analysing the nucleotide sequences of prominent RNase T1-resistant oligonucleotides obtained from complete digestion of internally radiolabelled RNAs *IFNA5* (197–446), (215–427) and (397–427) (Fig. 9). Electrophoretic analysis of RNase T1-resistant fragments revealed that the three RNAs contained a common, large and highly resistant fragment (bands size of s1 in Fig. 9a). Kinetic analysis of these RNase T1 digestion products indicated that the main resistant band for all three RNA fragments could be detected after 5 min of a 20 min reaction (data not shown).

Using the data concerning the length of the RNase T1 resistant products obtained from the 20 % polyacrylamide gels, together with identification of the secondary digestion products (Fig. S13), the majority of the sequence of protected fragment s1 was identified (51 out of approximately 59 bases were localised) and placed within the primary sequence of the *IFNA5* (329–427) RNA fragment (Fig. 9b), covering nearly all of domain D4. Thus providing evidence of this domain’s presence. The sequence for m3 was identified by the same process and found to be identical to s1 (data not shown).

### Structure probing of sequences carrying site-directed mutations

#### Four-branched structure

**Fig. 10:**
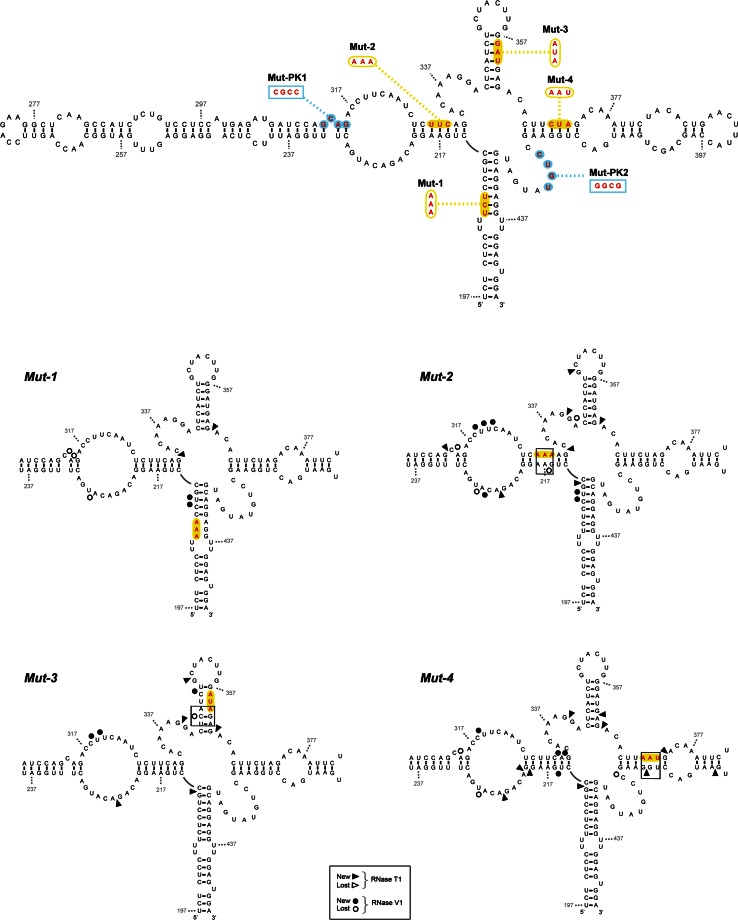
Secondary structure of *IFNA5* RNA (197–446) wt and Mut-1 to Mut-4 summarizing enzymatic probing. *Upper draw* secondary structure of *IFNA5* RNA (197–446) recompiling the location of the site-directed mutational changes in the individually analysed mutant sequences. The *yellow squares* indicate mutations of Mut-1 to Mut-4 sequences. The *blue circles* are mutations in PK1 and PK2 and the compensatory Mut-PK1+2 sequences. The nucleotide changes are provided above the wild-type sequence. Results from RNase T1 and RNase V1 partial digestion data from 5′ end-labelled *IFNA5* RNA (197–446) are represented by *arrowheads* and *circles*, respectively. Mut-1 to Mut-4 are depicted. Only new cleavage points or missing cleavages specific for each of the single mutant sequences in relation to wt are presented. Key changes in *boxes*. The results shown are from analysing 10 % as well as 6 % denaturing polyacrylamide gels

**Fig. 11:**
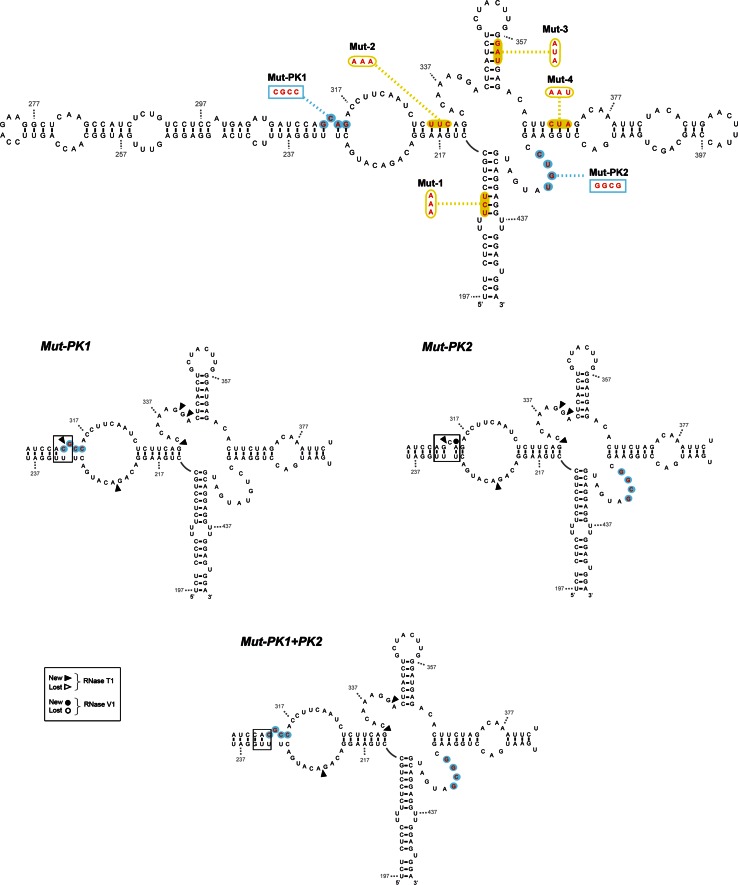
Secondary structure of *IFNA5* RNA (197–446) of PK mutant sequences summarizing enzymatic probing. RNase T1 and RNase V1 partial digestion data from 5′ end-labelled *IFNA5* RNA (197–446) are represented by *arrowheads* and *circles*, respectively. Mutant sequences Mut-PK1 and Mut-PK2 and restored Mut-PK1+2 are depicted. Key changes in *boxes*. The results shown are from analysing 10 % as well as 6 % denaturing polyacrylamide gels

Four mutant *IFNA5* (197–446), RNAs were employed to test whether predicted base pair disruptions did in fact alter the presumed four-branched structure. One mutant was generated for each branch, domains D1 to D4, and they were referred to as Mut-1, Mut-2, Mut-3 and Mut-4, respectively (see Fig. 10 for details). The structural effects of these base substitutions were assayed on 5′-[^32^P] end-labelled transcripts by RNases T1 and V1 digestions and compared with the wild-type cleavage pattern sequence (Figure S14 A and B). We grouped the observed alterations of RNase T1 and V1 patterns in Supplementary Table S2 according to whether a base had an altered reactivity in only one of the mutant sequences (specific mutant alteration) or whether alterations were repeated in more than one mutant sequence (structure-sensitive positions). Specific RNase cleavage alterations, which were placed in the opposite strand to the mutated positions, either provoked the appearance of a new RNase T1 cleavage (G_414_ for domain D4) or the disappearance of a RNase V1 cleavage (G_216_ and U_343_ for domains D2 and D3, respectively). These alterations supported the proposed D2, D3 and D4 domains in the helix junction region and are distinguished with larger letters in Supplementary Table S2 and represented in Fig. 11. The experiment yielded no information about domain 1. Other few specific reactivity alterations were present (Supplementary Table S2) but did not provide evidence of any further structures relevant to the wild-type sequence. These changes must be due to differences in the secondary or tertiary structure of the reorganized mutant RNAs that alter the nature of sites available for the nucleases in comparison with the wild-type molecule. It is also noticeable that the regions of *IFNA5* RNA more distant from the four helix junction, except G_405_ in Mut-4, are unchanged in their susceptibility to attack by RNases T and V1. This is a direct support for the stability for the end part of each domain and, indirectly for the central four-way junction configuration.

Additionally, changes in each mutant included at least a base reactivity alteration at the helix junction region of a different domain to that in which the mutation was generated (e.g. Mut-1 alters base G_332_ at D2–D3 junction; Mut-2 alters base G_363_ at D3–D4 junction; Mut-3 and Mut-4 alters base G_212_ at D1–D2 junction). Particularly, Mut-4 alters the junction position of the 3 other domains (Supplementary Table S2 and Fig. 11). Also the reactivity of bases in the sequences forming the predicted pseudoknot were altered. In most of these cases, the same position at the junction or the predicted pseudoknot was modified in different mutants, and so these positions belong to those previously classified as structure-sensitive. This result, probably due to destabilisation of the domains coaxial stacking and/or sterical facilitation of nucleases access to the central helix junction, greatly supported the proposed four-way junction structure.

#### Pseudoknot

The IPKnot program [62] predicted a pseudoknot between contiguous sequences (G_312_CAG_315_) and (C_317_CUU_320_), pairing with (C_420_UGU_423_) and (A_337_AGG_340_), respectively. We focused on the first pair of annealing sequences because it was across the junction and because bases (C_420_UGU_423_) within domain D4 lost three RNase V1 cleavage in RNA fragments lacking D2, as well as gained two Pb^2+^ cleavages and a DEPC cleavage at nearest neighbour A_424_, which could be a sign of interaction between the mentioned D4 bases and the complementary sequence in D2: G_312_CAG_315_, as predicted. Therefore, we analysed two individual PK mutants, referred to as Mut-PK1 and Mut-PK2, plus a third one with compensatory mutations, Mut-PK1+2 (Figs. 10, 11).

Changes in the probing pattern with RNase T1 in Mut-PK1 and Mut-PK2 (Fig. S14C) were constrained to position G_312_, at its neighbour sequence G_339_G_340_ and at G_224_ within the opposite strand. The single exception was position G_332_ in the helix junction region (Fig. 5). Alterations to RNase T1 cleavage at positions G_312_ and G_332_ were absent in complementary mutation Mut-PK1+2, indicating restoration of the pseudoknot structure across the junction studied. The altered RNase T1 positions and Gs: 224, 332, 340, (outside the pseudoknoted annealing sequences probed) in both Mut-PK1 and Mut-PK2 were not restored in the Mut-PK1+2, indicating that structural reorganization in the Mut-PK1+2 was somewhat distinct to the wt sequence. In the case of RNase V1, alterations of Mut-PK1 and Mut-PK2 sequences affected base positions A_314_ and C_318_UUC_321_, that were re-established in PK1+2.

In conclusion, results from site-directed mutants confirmed annealing between bases (C_420_UGU_423_) and (C_420_UGU_423_), previously predicted from nucleases as well as from chemical analysis of different fragment length RNAs, and lent support to the prediction of a contiguous pseudoknoted structure (coloured regions of structure shown in Fig. 7). In the resulting model, most of the positions previously qualified as displaying contradictory reactivities in RNA fragment (197–446): (A_221_C_222_) and (C_419_–G_422_), could be located within the pseudoknot.

#### Conformational analysis

Native 6 % non-denaturing polyacrylamide gels were used for conformational analysis of the *IFNA5* (197–446), (215–427) and (329–427) RNAs (Fig. S15). A major band was present in each of the three RNA fragments. The band corresponding to *IFNA5* (197–446) RNA was slightly wider than those in the other two fragments in repeated experiments. Parallel RNase T1 digestions were carried out to check whether the two larger fragments with different gel-band widths could also be distinguished by comparing nearby lanes containing the digests of RNA fragments. *IFNA5* RNAs (197–446) and (215–427) provided very similar patterns of partial digestion products (Fig. S16), indicating that they have similar structures, in keeping with the results obtained during probing experiments. Subtle differences between the two fragments were restricted to very weak bands (Fig. S16). These were interesting because they lay close to or within a flanking region of possible RNA:RNA interaction. The differences were a mild cleavage before A_316_ in the case of *IFNA5* (197–446) RNA, absent in the *IFNA5* (215–427) RNA fragment, and a mild cleavage before C_333_ in *IFNA5* (215–427) RNA, absent in the *IFNA5* (197–446) RNA fragment. The first difference was previously observed during structural probing (Figs. 7, 8); the second was not, but added to a region of two strong RNase A cleavage differences (i.e. C_333_ and C_335_).

### Regions determining human RNase P cleavage

**Fig. 12:**
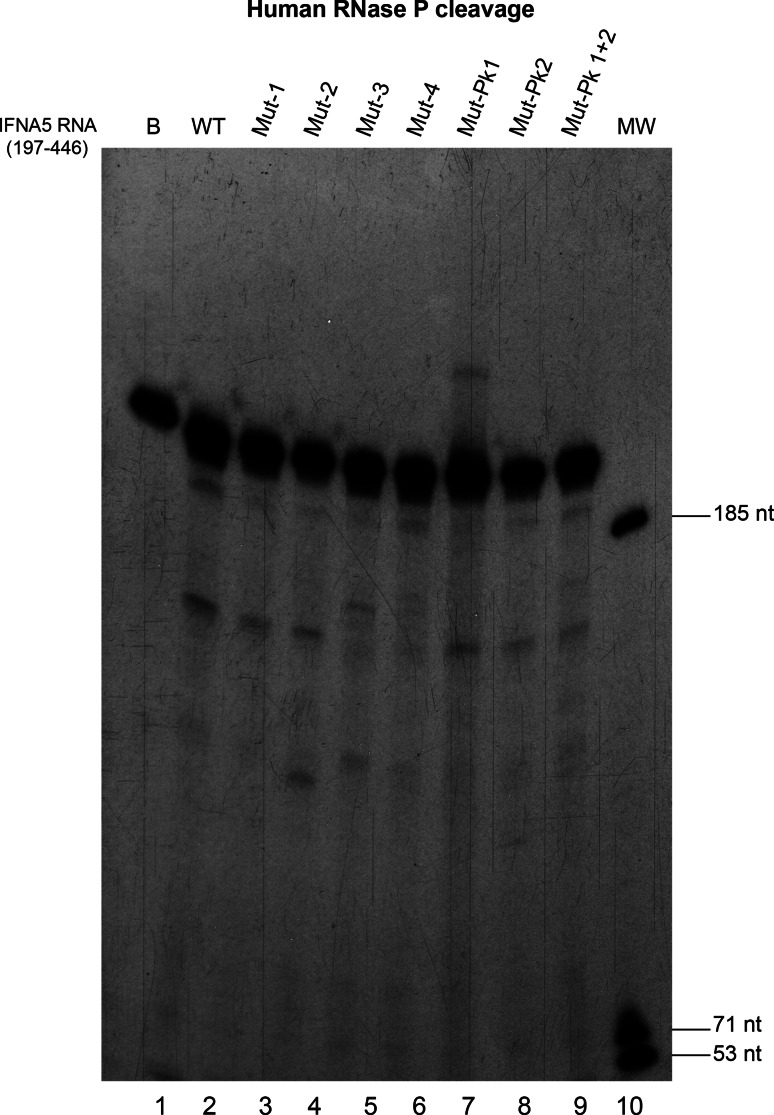
RNase P cleavage of *IFNA5* RNA (197–446) RNA site-directed mutants. Autoradiogram showing the RNase P specific cleavage of seven different *IFNA5* variants shown in this figure and wild-type RNA

To correlate the structural defects of the mutant sequences with requirements for human RNase P cleavage, the wt sequence was digested with RNase P in parallel to Mut and PK mutant sequences (Fig. 12). Two independent experiments provided the same electrophoretic cleavage pattern. Substitutions decreased the extent of cleavage of Mut-1 to Mut-3 mutants, altered cleavage specificity in Mut-2 and Mut-3—providing products of a slightly different length—and the cleavage was nearly abolished in D4. PK mutants were not affected severely.

### Similarities between IFNA5 and HCV tRNA-like motifs

When seeking parallels at the secondary structural level, the four-way junction regions of HCV RNA in basal stem loop III (see Fig. 7 of Lyons and Robertson [37]) [63] and the central core region of *IFNA5* RNA (Fig. 5), from which the four domains D1 to D4 emerge, participate in a pseudoknot across the helix junction region. In the primary structure, the presence of a CAR-E sequence in HCV positions C_292_–G_303_, although with a single insertion of two nts (ag) in the sequence (5′CCUGAUagGGUG3′) is remarkable. This is located in a structurally equivalent position to the *IFNA5* tRNA-like, in one of the branches and loops of the HCV tRNA-like domain.

## Discussion

### RNase P recognizable and cleavable sites are present in human liver mRNA

The use of enzymes or cell-extract preparations that specifically cleave structured RNA regions within eukaryotic hnRNA, viral and phage mRNA in vitro represented an important study tool in the field of gene-expression regulation in the 1970s and early 1980s. When such regions were found, they provided important information regarding the processing or function of the RNAs carrying them [64–67]. As this data began to emerge, it was shown that internally labelled hnRNA contained little or no target sites for RNase P from *E. coli* and HeLa cellular fractions [56, 68]. As these findings suggested that RNase P did not participate in any of the steps in which mature mRNAs were cleaved from longer primary transcripts, there seemed to be no role for RNase P in mature mRNA turnover/metabolism [56, 68]. Probably because of this fact, the specificity and proportion, if any, of RNase P-sensitive sites within eukaryotic mRNA species remains unknown.

The primary objective of the experiments described in this study was to explore whether tRNA-like structures reported for many viral RNA genomes exist in host mRNAs. The results of our human RNase P competition assays indicated the presence of RNase P recognizable structures within the mRNA population, but direct examination of the incubation products of mRNA with RNase P suggested that most mRNA species do not contain cleavable RNase P sites. This is in agreement with published results on the 1970s [43, 52]. Subsequently, low but reproducible signal values for cleavage were observed for the highly purified *Synechocystis* sp. RNase P ribozyme through microarray screening in order to detect individual species. Three mRNAs selected from the screening were also found to be specific substrates for the human RNase P enzyme. For both RNase P activities and for the three RNAs tested, the proportion of cleavage products of the RNA which entered into the reaction were around 10 % or less. This low cleavage percentage might provide an explanation for the difference between the competition and cleavage experiments in mRNA populations, indicating that most elements with RNase P recognizable structural properties within mRNA are neither stable nor perfectly shaped, while also being cleavable. Indeed, the presence of an enzyme that can cleave mRNAs in the nucleus of the eukaryotic cell should have represented an evolutionary constraint by favouring imperfect or unstable tRNA-mimic structures rather than perfect mimicking of cleavable motifs. Under this assumption, the frequency of tRNA-like motifs within mRNA cannot be estimated from our results. All that can be concluded is that RNA structural elements carrying features of tRNA are present within mRNAs; they may occupy different locations within the message chain and may be present more than once in the same molecule. However, whatever the specific role of these RNase P-sensitive structures built into mRNA, their biological importance for molecular communication can be hypothesized.

### The example of IFNA5 mRNA

We have focused our study on interferon-alpha subtype 5 mRNA as it is a liver-specific variant [69] that shows the same tropism as HCV. In this particular case, RNase P cleavage specificity was further determined by the precision of the cleavage site in sequencing analysis and by characterising newly generated end-groups after 5′-P and 3′-OH cleavage, which are characteristic cleavages of RNase P [12].

### The cloverleaf structure

Nuclease probing results from both of the labelled ends of three different fragment length RNAs showed a high level of mutual agreement and provided strong evidence of a secondary structure similar to a cloverleaf (Fig. 5). A small number of contradictory reactivities were found, i.e. bases that are simultaneously reactive to both paired and unpaired probes or which do not concur with the predicted secondary structure. Only four of these cases were considered especially relevant: RNase A at C_397_ could be explained by ‘breathing’ of the short stem in which C_397_ is located; RNases V1 at A_221_–C_222_, U_305_ and U_437_, were located on internal loops between large dsRNA regions and thus, do not seem to indicate a structure difference to that which is proposed. In particular, the dsRNA region flanking the internal loop where U_305_ resides was determined to be a substrate for specific double-stranded nuclease *E.coli* RNase III (Díaz-Toledano et al. in preparation). This lends strong support to the idea that the altered base reactivity does not represent a distortion of the structure proposed for domain D2. RNase V1 also cuts at base stacked positions, and this could be an explanation of RNase V1 cleavages in apparent internal loops [53]. RNase V1 cleavages at positions (C_419_–G_422_) are considered differently (see “Pseudoknot” section). Chemical analysis also supported the proposed structure.

The main conclusion from the structural analysis of the three fragment lengths were the general similarity of the nuclease mapping within the common sequence. Deletion of D1, or D1 and D2, had little effect on the rest of the structure thus indicating that the predictions were accurate for these two domains. The most notable differences between fragments D2–D3–D4 and D3–D4 are due to the presence of the pseudoknot.

Additional support for the structure proposed came from the RNase T1 protection experiments. We observed a strongly protected sequence approximately 59 nts in length and which included nearly the whole of domain 4 and seven “G” residues. The failure of ribonuclease T1 to cleave after this number of “G” bases in nuclease saturated reactions, irrespective of the size of RNA being analysed, implies that this region has a self-protecting fold, providing strong evidence that D4 is one of the domains.

Finally, in a mutational study, new RNase cleavages correlated with specific changes in the opposite chain of the secondary structure model for domains D2, D3 and D4 in the junction region, thus in agreement with the proposed structure. D1 analysis was uninformative.

Of the additional alterations that were observed, most were classified as structure-sensitive positions either grouped in the four-way junction or in the predicted pseudoknot. In the four-way junction, mutation of each domain could generate changes in other domains, probably due to destabilisation of helices coaxial stacking and/or sterical obstructions; this could facilitate the attack of nucleases at the central helix junction. Bases G_212_, G_332_, G_363_, on the D1–D2, D2–D3 and D3–D4 junctions, respectively, had modified RNase T1 cleavage patterns; again, a strong indicator supporting the cloverleaf structure and in turn the four-way junction structure.

Regarding RNase P determinant motifs, RNase P cleavage locates at position A_377_↓A_378_ and thus within the *IFNA5* (329–427) RNA fragment, containing domain D3–D4, but a larger fragment containing D2–D3–D4 is required for cleavage. This it implies that the sequences/structures flanking domain D3–D4 should have relevant roles in specifying human RNase P recognition. Domain 2 provides a third helix to the cloverleaf structure. Mutations of each domain also indicated that D4 stem disruption had the worst effects on cleavage reactivity. D2 and D3 stems also contributed to recognition as their modification changed cleavage specificity positions and decreased the extend of the processing reaction. The pseudoknot was not essential for cleavage. In comparison to RNase P minimal substrate, domain 3 would “represent” the T-stem and T-loop of tRNA and domain 4 the acceptor stem [70].

The pseudoknot in *IFNA5* might be responsible for the adequate orientation of the branches, as in the tRNA-like structure in TMV, where the helix junction is integrated within a pseudoknot [58] although that is a three-way junction element.

### Conformational analysis

Native gels (Supplementary Fig. 15) showed a major band for the different transcripts indicating the presence of a single conformation or, at least, very similar ones. The fact that the band for the larger *IFNA5* (197–446) RNA fragment was broader than those of *IFNA5* RNAs (329–427) and (215–427) RNAs suggests that this RNA fragment is breathing at some key structural sites. This interpretation was supported by the results from a parallel RNase T1 probing (Supplementary Fig. 16) that revealed greater accessibility of G_315_ which favours the idea of a subtle instability of the RNA:RNA interaction forming the pseudoknot in the larger *IFNA5* (197–446) RNA fragment.

### IFNAs CAR signal

A CAR signal, which is able to interact with factors for nuclear export and cytoplasmic stabilisation, has been characterised within a conserved secondary structure region, in both *IFNA1* and *IFNB1* mRNAs [71]. When the exact positions are extrapolated to *IFNA5* RNA, the regions occupied by CAR correspond to positions 242–424. This region coincides significantly with the minimal region required for RNase P cleavage, namely *IFNA5* RNA (215–427). Functional CAR signals involve primary sequence elements (one to four) 10 nts in length, referred to as CAR-E [72]. Here we determined that each CAR-E is located in the extremity of domains 2 and 4 of the tRNA-like motif, positions C_264_–G_273_ and C_396_–G_405_, highlighted in grey in Fig. 5. Apart from this positional symmetry, CAR-E adopts an equivalent secondary structure.

### Similarities between IFNA5 and HCV tRNA-like motifs

It is possible that the similarly placed recognition elements for the human RNase P enzyme in these viral 5′ UTR domains including HCV, CSFV, BVDV and cellular *IFNA*s mRNA, together with key consensus sequence, structural similarities, and unconventional polarity of cleavage by RNase P reflect the presence of a similar RNA signal for the molecules that carry them. In these line of argument, the common presence of Aly protein bound to 5′ UTR of HCV [73] and to *IFNA* CAR [71] might provide a clue of the functional correlate for the structural mimicry found between viral and host elements. Nevertheless, further experiments will be needed to prove this hypothesis.

Whatever it means for molecular communication, our results open the door to examining whether any of the mRNA species identified could provide clues about the advantages to the viruses of incorporating tRNA-mimicking elements in their genomes, and in particular whether or not HCV RNA, and other viruses that contain such elements in their genome, acquire properties from the host “CAR” mRNA signal.

## Electronic supplementary material


**Figure S1**: Visualization of the human liver mRNA population after cleavage by RNase P activities. Digestion of 350 ng of mRNA with human RNase P (panel A) or *Synechocystis sp*. ribozyme (panel B) was analysed by automated electrophoresis techniques under denaturing conditions using an Experion™ (Bio-Rad). **A**) Human RNase P electropherograms: the light blue line corresponds to mRNA digestion with human RNase P for 30 min (R1), the green line to the same reaction for 60 min (R2) and the dark blue line to a control run of the mRNA incubated in buffer for 60 min followed by addition of a 0.5 µL aliquot of RNase P extract at the same time as the proteinase K and SDS % inactivation at the end of the incubation. This control was performed to evaluate the presence of RNAs contained in the RNase P extract, which were considered to be negligible. **B**) *Synechocystis sp.* electropherograms: red line corresponds to human liver mRNA incubated on ice (commercial sample), green line 60 min reaction (R), dark blue mRNA incubated on buffer (B) (TIFF 11634 kb)

**Figure S2**: Summary and schematic representation of the screening analysis and interpretation for RNase P target mRNAs. The commercial microarrays employed (20 K Human Genome G4110B-Agilent technologies) were originally designed for gene-expression evaluation. In our experimental approach, a straightforward extrapolation of the concept of over/under-expression to cleaved/uncleaved mRNAs might be unsuitable. The results are interpreted according to the two alternate hypotheses depicted schematically in the figure. First, it should be noted that most oligonucleotides that act as gene probes fixed in the microarray support (red) are complementary to the 3′ coding region of the mRNAs. If the RNase P (represented as a “man with scissors”) cleavage site is localised upstream of the probe hybridization region (panel **A**), the cleaved RNA generates a shorter template that still carries the probe hybridization region and would therefore be expected to be detected with similar intensity as the full length mRNA in the non-cleaved control essay. However, the cleaved and shortened substrate with a reduced degree of structure may have made it easier for the reverse transcriptase enzyme to copy it into its corresponding cDNA, thus providing a higher amplification efficiency. When this effect occurs, the hybridization signal would be expected to be stronger than the control one. In the case that RNase P cleaves between the poly(A) tail and the target region (panel **B**), this would imply a direct decrease of the signal being analyed. Selected mRNA species were clustered using the program Panther Applied in order to find groups with similar activity patterns. Despite being widely dispersed as regards the biological function encoded, several mRNA species grouped within the area of primary metabolism (human RNase P: 11; *Synechocystis sp*. ribozyme: 23) and, to a lesser extent, in the areas of nucleic acid metabolism, cell communication, development processes and the immune system. The mean fold changes obtained in the duplicate experiment were: for *IFNA5* mRNA: -1.22 and -1.35 with Brank 98,05 % and 91,60 %; for *H2AFJ* mRNA: 1.51 and 1.53 with a Brank 95.55 % and 91.93 % and, for *RPS9* mRNA: 1.51 and 1.82 with Brank 95.98 % and 95.15 % (TIFF 12936 kb)

**Figure S3**: Three selected mRNA species are processed in a dose-dependent manner by *Synechocystis sp.* RNase P ribozyme. All transcripts were internally radiolabelled during *in vitro* transcription and incubated with increasing amounts of *Synechocystis sp.* ribozyme. In all cases: lane 1 RNA incubated on ice and lane 2 incubated in the ribozyme reaction buffer. **A**) *IFNA5* RNA (1-700): lanes 3-7 reactions with the ribozyme at a concentration of 33.75 nM, 67.5 nM, 135 nM, 270 nM and 540 nM, respectively. Lane 8: molecular weight markers. Arrows in the left indicate band products P1′ and P7′ corresponding to human RNase P products P1 and P7. **B**) *H2AFJ* RNA (1-658): lanes 3-8 reactions with the ribozyme at a concentration of 33.75 nM, 67.5 nM, 135 nM, 270 nM, 540 nM and 675 nM. **C**) *RPS9* RNA (1-714): same as panel B (TIFF 20046 kb)

**Figure S4**: Mapping the cleavage sites in *H2AFJ* and *RPS9* mRNAs. Panels **A** and **B**) Autoradiogram of human RNase P cleavage of internally radiolabelled (lanes 1-3), 5′- (lanes 4-6) and 3′-end-labelled (lanes 7-9) *H2AFJ* and *RPS9* RNA transcripts, respectively. Lanes 1, 4 and 7: RNA incubated on ice; lanes 2, 5 and 8: RNA incubated in reaction buffer; lanes 3, 6 and 9: reactions with human RNase P. Lane 10 is a molecular weight ladder. Arrows on the left side indicate the major digestion products designated as H1 to H4 for *H2AFJ *RNA and S1 and S2 for *RPS9* RNA. The 5′-end-labelled product gave two bands, H1 and H2, thus indicating that the RNA was cleaved at two nearby sites between the coding and 3′ non-coding region. In contrast, only a single intense cleavage product (H3), referred to herein in the main text as primary cleavage, was observed from the 3′-end-labelled RNA. This product band (H3), and its partner product band H1, comprise the total 1-658 base transcript. Band H2 could be the result of an additional cleavage of H1 by RNase P, which does not occur in the entire 1–658 RNA substrate (secondary cleavage). Panels **C** and **D**) Linear diagrams of the *H2AFJ* substrate transcript (1-658 nt) and *RPS9* (1-714) segmented every 100 nts. Grey areas indicate the protein-coding region and white areas the untranslated flanking regions. The final cleavage products were deduced from the bands observed on a 4 % polyacrylamide electrophoresis gels and are represented by arrows (TIFF 7633 kb)

**Figure S5**: Determination of human RNase P minimal substrate and cleavage specificity for* H2AFJ *and *RPS9 *mRNAs. Internally labelled RNAs of *H2AFJ* (panel A) and *RPS9* (panel B and C) were incubated in reaction buffer (lanes 2 and 6), in the presence of human RNase P (lanes 3 and 7), with addition of unlabelled pre-tRNA^Tyr^ (10 × molar excess) (lanes 4 and 8), or poly-r(A) (equivalent quantity by weight) (lanes 5 and 9) respectively. Lane 1 shows the century molecular markers. *RPS9* (8-326) and (27-215) RNAs, incubation with human RNase P activity provided a product approximately 180 nts in length (Sup. Figure 7B) derived from a new cleavage site (not found in the full length *RPS9* RNA (1-714) (Fig. 2C). This new product disappeared in the shortened *RPS9* (159-215) fragment (Sup. Figure 7C), thereby probably indicating an altered structure in the shortened *RPS9* transcripts (TIFF 8459 kb)

**Figure S6**: Biochemical characterization of *IFNA5* RNase P cleavage product end-groups. *IFNA5* (1-700) was labelled at a sufficiently low specific radioactivity (10^5^ dpm/µg) in order to trace its electrophoretic mobility and to permit an increase in radioactivity incorporated in the subsequent end-labelling reactions. RNase P digestion product bands P1, P3, P5 and P7 (see Fig. 2 panel A) were purified by preparative gel electrophoresis and subjected to different specific enzymatic treatments to determine the chemical groups. A) Determination of the newly generated 5′-ends by T4 RNA ligase circularization. Lane 1: molecular weight; lane 2: *IFNA5* RNA subjected to a standard human RNase P reaction; lanes 3 and 5: product bands P5 and P7 incubated on ice; lanes 4 and 6: product bands P5 and P7 incubated with the enzyme. New bands in the T4 RNA ligase treatment with delayed mobility than the untreated material, corresponded to circularized P5 and P7, thus confirming their 5′-P end. B) Enzymatic determination of new 3′-end by labelling with T4 RNA ligase and [^32^P]pCp. Lanes 1 and 3: bands P1 and P3 incubated on ice; lanes 2 and 4: P1 and P3 in presence of the enzyme and [^32^P] pCp. Increase of label in the presence of enzyme and [^32^P]pCp is indicative of 3′-OH end in P1 and P3. C) Determination of the newly generated 5′-ends by differential phosphatase/kinase and [γ-^32^P]ATP treatment. Lane 1: molecular weight ladder; lane 2: *IFNA5* RNA subjected to a standard human RNase P reaction; lanes 3 and 6: bands P5 and P7 incubated on ice; lanes 4 and 7: bands P5 and P7 labelled with T4 polynucleotide kinase and [γ-^32^P]ATP after prior incubation with phosphatase; lanes 5 and 8: treated identically as before but without alkaline phosphatase pretreatment. A characteristically low increase in label was observed in the phosphatase-treated samples, thus indicating the presence of a 5′-P (TIFF 8256 kb)

**Figure S7**: Determination of human RNase P cleavage sites on *IFNA5* mRNA by indirect sequencing. A) The table summarizes the results obtained upon subcloning and sequencing of cleavage product bands P1, P3, P5 and P7. The number of clones sequenced and the frequency of representation of clones for each cleavage position is described for each product. B**)** Positioning of the two most represented RNase P cleavage sites on the predicted secondary structure model for *IFNA5* mRNA (using *Mfold* program). Arrow lengths are proportional to the observed frequency of each cleavage position (TIFF 5555 kb)

Direct determination of human RNase P cleavage sites in* IFNA5* by direct sequence analysis. Both transcripts were labelled at its 3′ end with [^32^P]pCp. *IFNA5* (197–446) RNA: lane 1 RNA transcript alone incubated in buffer Arrow indicate the most prominent band (P1). Samples were electrophoresed on a 6 % denaturing polyacrylamide gels (TIFF 4082 kb)

**Figure S9**: Enzymatic probing of the secondary structure of *IFNA5* RNA (215-427). Panels A and B: 5′-[^32^P] end-labelled RNA. Panels B and C: 3′-[^32^P] end-labelled RNA. For all cases: lane 1 alkaline hydrolysis reaction (OH); lane 2 RNase T1 reaction under denaturing conditions (T1L); lane 3 RNase T1 (T1), lane 4 RNase V1 (V1) and lane 5 RNase A (A) under standard conditions, respectively. Denaturing gels were at 10 % (panels A and C) or 6 % polyacrylamide (panels B and D) (TIFF 8802 kb)

**Figure S10**: Enzymatic probing of the secondary structure of *IFNA5* (329-427). Panels A and B: 5′-[^32^P] end-labelled RNA. Panels B and C: 3′-[^32^P] end-labelled RNA. For all cases: lane 1 RNA incubated on ice (I); lane 2 alkaline hydrolysis reaction (OH); lane 3 RNase T1 reaction under denaturing conditions (T1L); lane 4 RNase T1 (T1), lane 5 RNase V1 (V1) and lane 6 RNase A (A) under standard conditions, respectively. Denaturing gels were at 10 % (panels A and C) or 6 % polyacrylamide (panels B and D) (TIFF 7101 kb)

**Figure S11**: DEPC probing of 3′ end-labelled *IFNA5* RNAs (215-427) and (329-427). Panels A and B: 3′-[^32^P] end-labelled RNA (215-427). Panels C and D: 3′-[^32^P] end-labelled RNA (329-427). Lane 1 is the RNA maintained on ice (I). Lanes 2 and 3 are the products from digestion with alkali (OH) and RNase T1 under denaturing conditions (T1L), respectively. Products of aniline-treated RNAs previously modified with DEPC under native conditions (lane 4), semi-denaturing conditions (lane 5) and denaturing conditions (lane 6). Denaturing gels were at 10 % (panels A and C) or 6 % polyacrylamide (panels B and D) (TIFF 8827 kb)

**Figure S2**: Pb^2+^ probing of 3′ end-labelled I*FNA5 *RNAs (215-427) and (329-427): Panels A and B: 3′-[^32^P] end-labelled RNA (215-427). Panels C and D: 3′-[^32^P] end-labelled RNA (329-427). Lane 1 is the RNA maintained on ice (I). Lane 2 treatment with alkali (OH); lane 3 treatment with RNase T1 under denaturing conditions (T1L); lane 4 treatment with Pb^2+^ at 4ºC for 15 min and lane 5 treatment with Pb^2+^ at 30ºC for 20 s. Denaturing gels were at 10 % (panels A and C) or 6 % polyacrylamide (panels B and D) (TIFF 9059 kb)

**Figure S13**: Identification of complete RNAse T1 digestion products of *IFN5A* RNA (329-427) A: Sequence of the RNA markers used. 19 to 26 bases oligonucleotides sequences from RNA (329-427) employed as RNA precursors which are cleaved with RNase T1. The reaction yield a subset of short oligonucleotides (indicated by arrows, and numbered) for use as mobility controls in a high percentage denaturing polyacrylamide gel. B: The partial digestion products of [^32^P]-labelled *IFNA5 *RNA (329-427) S1 to S4 (lanes 1 to 4) were re-digested to completion with RNase T1 (lanes 5 to 8), and the products analysed in the presence of known subsets of the expected complete products (produced from the oligonucleotides described in panel A) (lanes 10-15) on 26 % denaturing polyacrylamide gel. The numbers within the gel identify the deduced band sequences described in panel A. Oligonucleotides used for mobility markers were either “G” or “U” labelled to facilitate identification. “U” labelled oligonucleotide “a” was lost during purification. C: 20 % denaturing polyacrylamide gel showing the mobility of products S1, S2, S3 and S4 (lanes 2-5) in comparison with “decade” RNA molecular weight markers (lane 1) (TIFF 8137 kb)

**Figure S14**: Enzymatic probing of *IFNA5* RNA (197-446) mutants. 5′-[^32^P] end labelled was employed for all RNA substrates. Panels A and B: Domains 1 to 4 mutants. Panel C: Pseudoknot mutants. The detailed sequences of each mutant were described in Fig. 12 of the main text. Panel A (RNase T1 reactions) and B (RNase V1 reactions): lane 1 alkaline hydrolysis reaction (OH); lane 2 RNase T1 under denaturing conditions (T1L); lanes 3 to 7 corresponded to Wt, Mut-1 to Mut-4 sequences, respectively. Panel C: lanes 1-4 (RNase T1) and lanes 5 to 8 (RNase V1) incubation of Wt sequence, Mut-Pk1, Mut-Pk2 or restoring mutant Mut-Pk1+2, respectively. Denaturing gels were at 10 % polyacrylamide. The numbers on the left indicate the point of digestion cleaved by RNase T1 under denaturing conditions (T1L), as identified with the help of the OH^−^ sequence ladder (TIFF 4645 kb)

**Supplementary Fig. 15**: Native gel of [^32^P] internally labelled* IFNA5 *RNA fragments. Autoradiography of non-denaturing 6 % polyacrylamide gel of ^32^P-labelled RNA samples. RNAs were denatured at 90ºC in water and left to cool in standard buffer. Each run was performed overnight at 4ºC. Lane 1: (197-446); lane 2: (215-427); lane 3: (329-427) (TIFF 1636 kb)

**Supplementary Fig. 16**: Comparative RNase T1 pattern of RNAs (197-446) and (215-427). 3′ end-labelled *IFNA5* (197-446) RNA (lanes 1-3) and *IFNA5* (215-427) RNA (lanes 4-6) were subjected to parallel RNase T1 digestion and run in 6 % denaturing polyacrylamide gel. RNAs were treated with alkali (lanes 1 and 4), RNase T1 under denaturing conditions (lanes 2 and 5) and RNase T1 under native conditions (lanes 3 and 6). The numbers on the right indicate the point of digestion cleaved by RNase T1 under denaturing conditions (T1L), as identified with the help of the OH sequence ladders (TIFF 17839 kb)

Supplementary Table S1 (PDF 57 kb)

Supplementary Table S2 (PDF 101 kb)
