# Supplementary figures and images for: Messenger RNAs bearing tRNA-like features exemplified by interferon alfa 5 mRNA
Source: Cell Mol Life Sci. 2015 Apr 22;72(19):3747–68. doi: 10.1007/s00018-015-1908-0 (PMC4565877; doi:10.1007/s00018-015-1908-0)

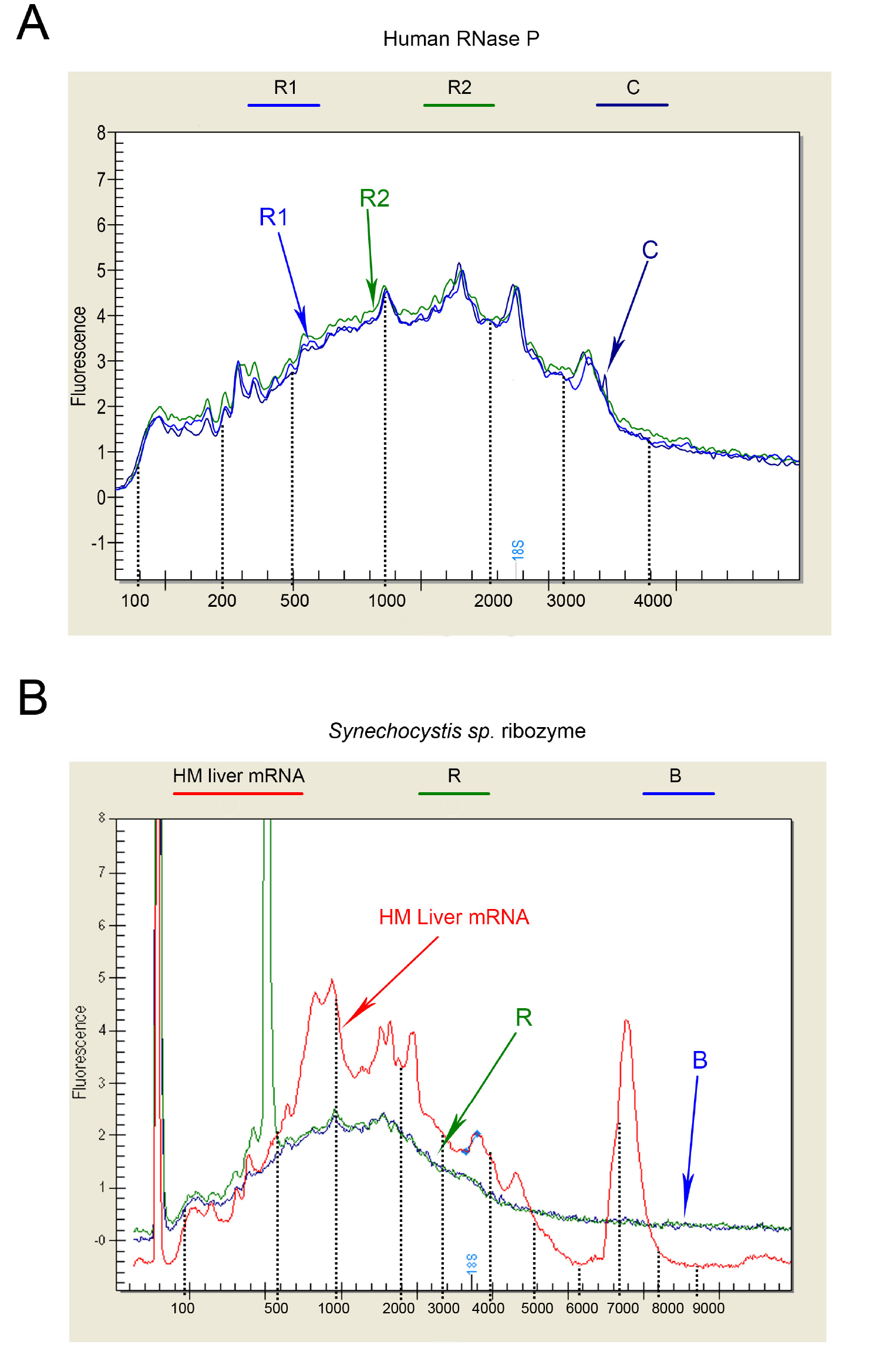

Supplement: Supplementary file 1 — Figure S1: Visualization of the human liver mRNA population after cleavage by RNase P activities. Digestion of 350 ng of mRNA with human RNase P (panel A) or Synechocystis sp. ribozyme (panel B) was analysed by automated electrophoresis techniques under denaturing conditions using an Experion™ (Bio-Rad). A) Human RNase P electropherograms: the light blue line corresponds to mRNA digestion with human RNase P for 30 min (R1), the green line to the same reaction for 60 min (R2) and the dark blue line to a control run of the mRNA incubated in buffer for 60 min followed by addition of a 0.5 µL aliquot of RNase P extract at the same time as the proteinase K and SDS % inactivation at the end of the incubation. This control was performed to evaluate the presence of RNAs contained in the RNase P extract, which were considered to be negligible. B) Synechocystis sp. electropherograms: red line corresponds to human liver mRNA incubated on ice (commercial sample), green line 60 min reaction (R), dark blue mRNA incubated on buffer (B) (TIFF 11634 kb) [file 18_2015_1908_MOESM1_ESM.tif]

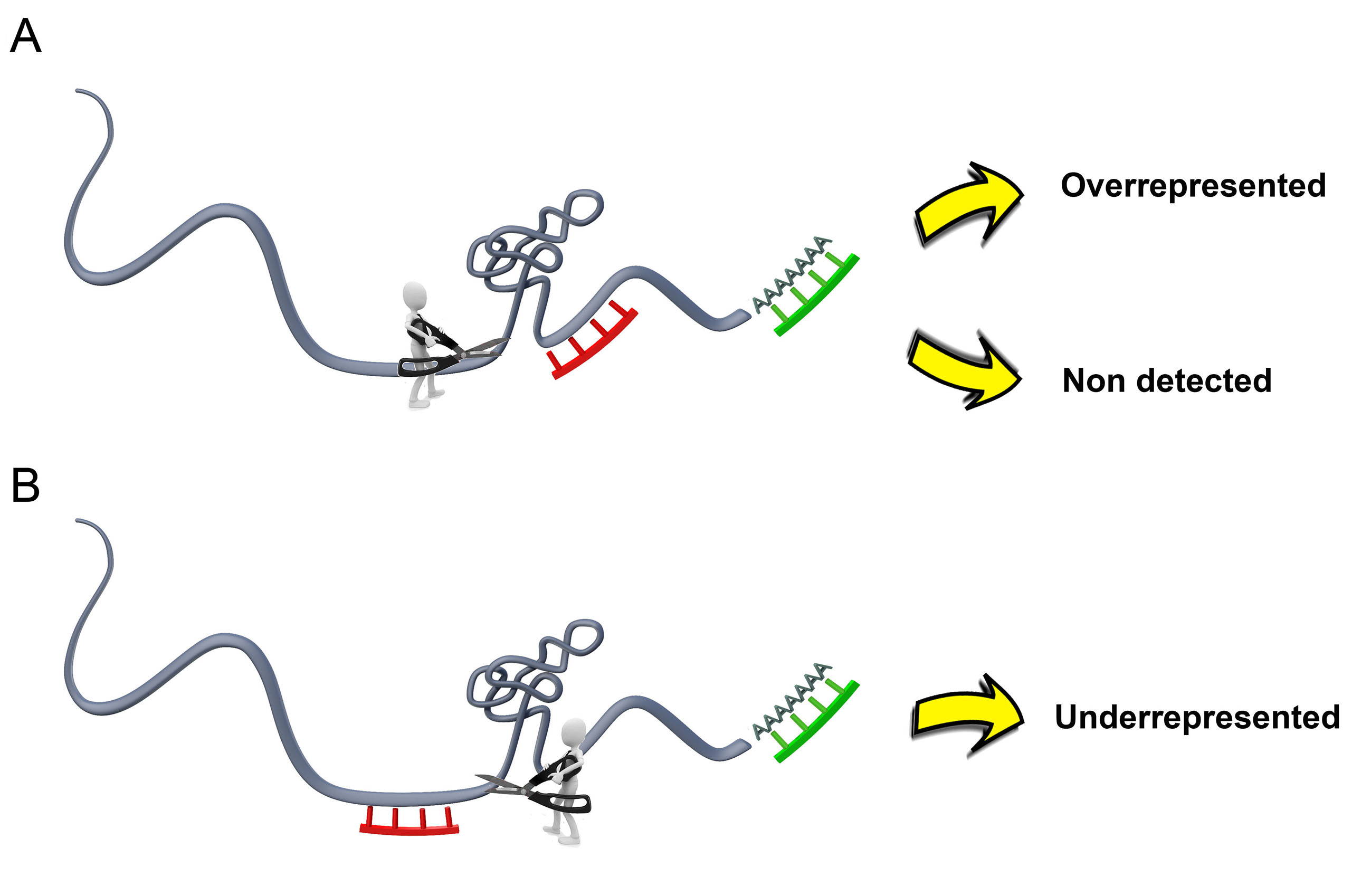

Supplement: Supplementary file 2 — Figure S2: Summary and schematic representation of the screening analysis and interpretation for RNase P target mRNAs. The commercial microarrays employed (20 K Human Genome G4110B-Agilent technologies) were originally designed for gene-expression evaluation. In our experimental approach, a straightforward extrapolation of the concept of over/under-expression to cleaved/uncleaved mRNAs might be unsuitable. The results are interpreted according to the two alternate hypotheses depicted schematically in the figure. First, it should be noted that most oligonucleotides that act as gene probes fixed in the microarray support (red) are complementary to the 3′ coding region of the mRNAs. If the RNase P (represented as a “man with scissors”) cleavage site is localised upstream of the probe hybridization region (panel A), the cleaved RNA generates a shorter template that still carries the probe hybridization region and would therefore be expected to be detected with similar intensity as the full length mRNA in the non-cleaved control essay. However, the cleaved and shortened substrate with a reduced degree of structure may have made it easier for the reverse transcriptase enzyme to copy it into its corresponding cDNA, thus providing a higher amplification efficiency. When this effect occurs, the hybridization signal would be expected to be stronger than the control one. In the case that RNase P cleaves between the poly(A) tail and the target region (panel B), this would imply a direct decrease of the signal being analyed. Selected mRNA species were clustered using the program Panther Applied in order to find groups with similar activity patterns. Despite being widely dispersed as regards the biological function encoded, several mRNA species grouped within the area of primary metabolism (human RNase P: 11; Synechocystis sp. ribozyme: 23) and, to a lesser extent, in the areas of nucleic acid metabolism, cell communication, development processes and the immune system. The mean f [file 18_2015_1908_MOESM2_ESM.tif]

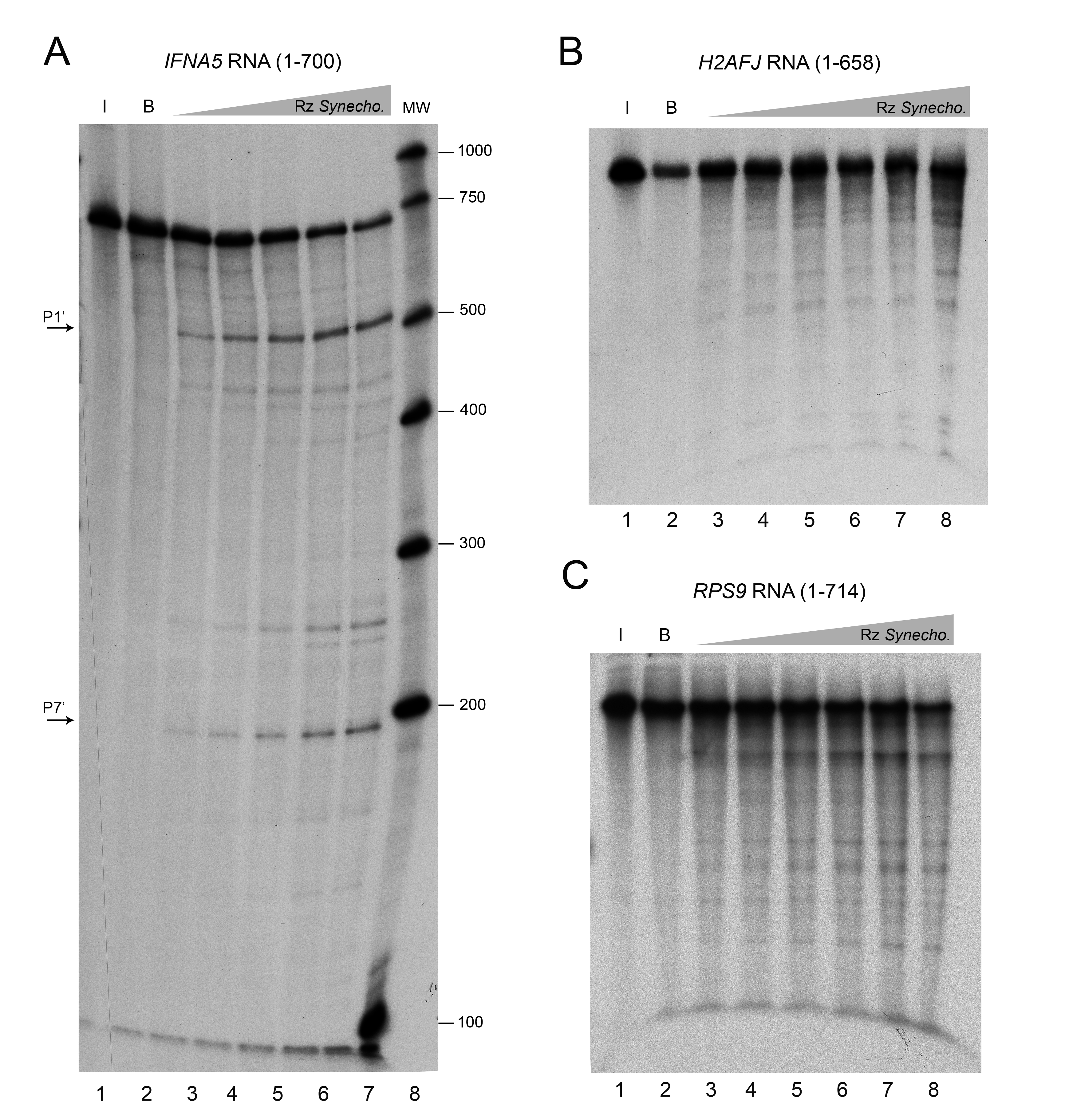

Supplement: Supplementary file 3 — Figure S3: Three selected mRNA species are processed in a dose-dependent manner by Synechocystis sp. RNase P ribozyme. All transcripts were internally radiolabelled during in vitro transcription and incubated with increasing amounts of Synechocystis sp. ribozyme. In all cases: lane 1 RNA incubated on ice and lane 2 incubated in the ribozyme reaction buffer. A) IFNA5 RNA (1-700): lanes 3-7 reactions with the ribozyme at a concentration of 33.75 nM, 67.5 nM, 135 nM, 270 nM and 540 nM, respectively. Lane 8: molecular weight markers. Arrows in the left indicate band products P1′ and P7′ corresponding to human RNase P products P1 and P7. B) H2AFJ RNA (1-658): lanes 3-8 reactions with the ribozyme at a concentration of 33.75 nM, 67.5 nM, 135 nM, 270 nM, 540 nM and 675 nM. C) RPS9 RNA (1-714): same as panel B (TIFF 20046 kb) [file 18_2015_1908_MOESM3_ESM.tif]

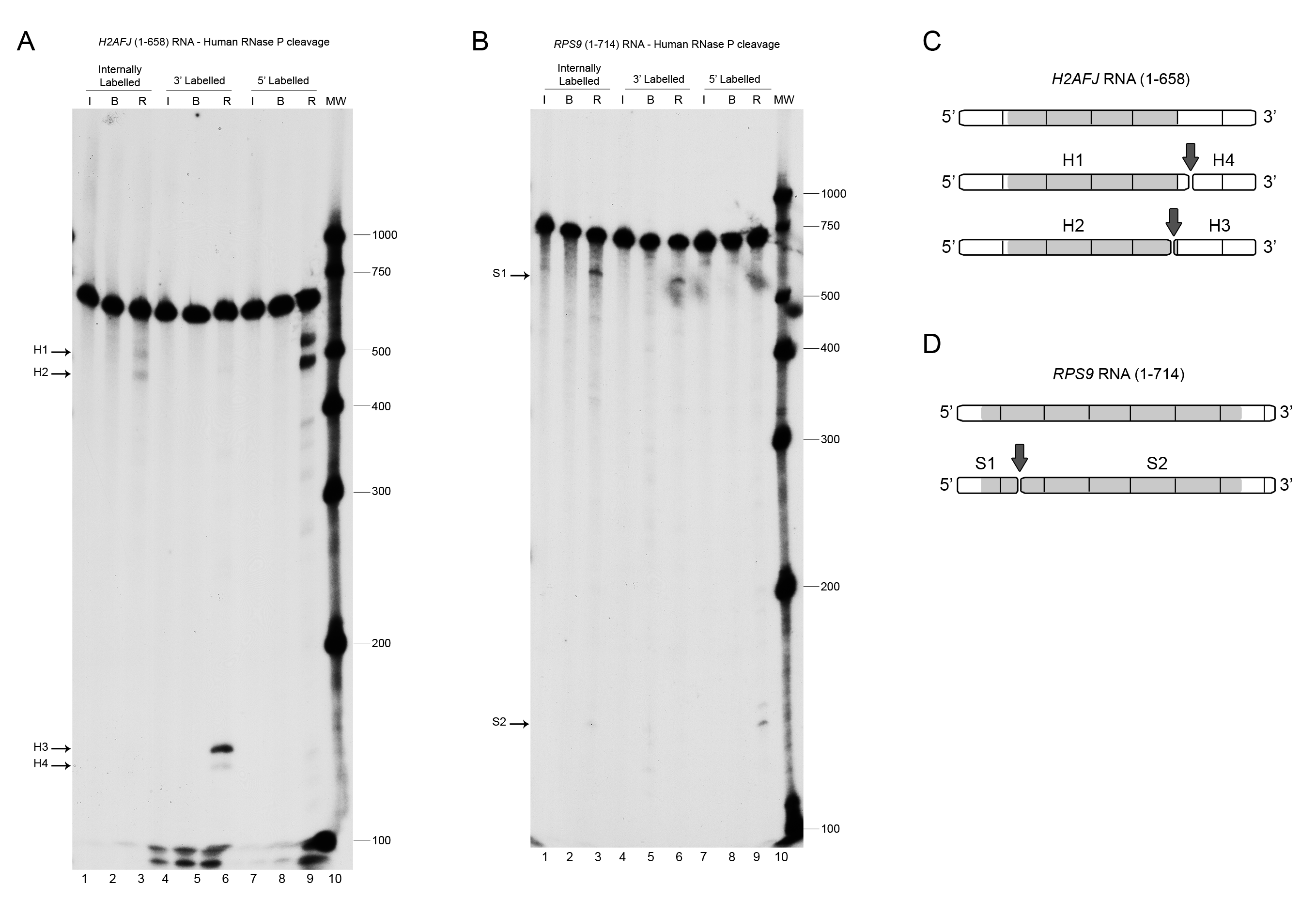

Supplement: Supplementary file 4 — Figure S4: Mapping the cleavage sites in H2AFJ and RPS9 mRNAs. Panels A and B) Autoradiogram of human RNase P cleavage of internally radiolabelled (lanes 1-3), 5′- (lanes 4-6) and 3′-end-labelled (lanes 7-9) H2AFJ and RPS9 RNA transcripts, respectively. Lanes 1, 4 and 7: RNA incubated on ice; lanes 2, 5 and 8: RNA incubated in reaction buffer; lanes 3, 6 and 9: reactions with human RNase P. Lane 10 is a molecular weight ladder. Arrows on the left side indicate the major digestion products designated as H1 to H4 for H2AFJ RNA and S1 and S2 for RPS9 RNA. The 5′-end-labelled product gave two bands, H1 and H2, thus indicating that the RNA was cleaved at two nearby sites between the coding and 3′ non-coding region. In contrast, only a single intense cleavage product (H3), referred to herein in the main text as primary cleavage, was observed from the 3′-end-labelled RNA. This product band (H3), and its partner product band H1, comprise the total 1-658 base transcript. Band H2 could be the result of an additional cleavage of H1 by RNase P, which does not occur in the entire 1–658 RNA substrate (secondary cleavage). Panels C and D) Linear diagrams of the H2AFJ substrate transcript (1-658 nt) and RPS9 (1-714) segmented every 100 nts. Grey areas indicate the protein-coding region and white areas the untranslated flanking regions. The final cleavage products were deduced from the bands observed on a 4 % polyacrylamide electrophoresis gels and are represented by arrows (TIFF 7633 kb) [file 18_2015_1908_MOESM4_ESM.tif]

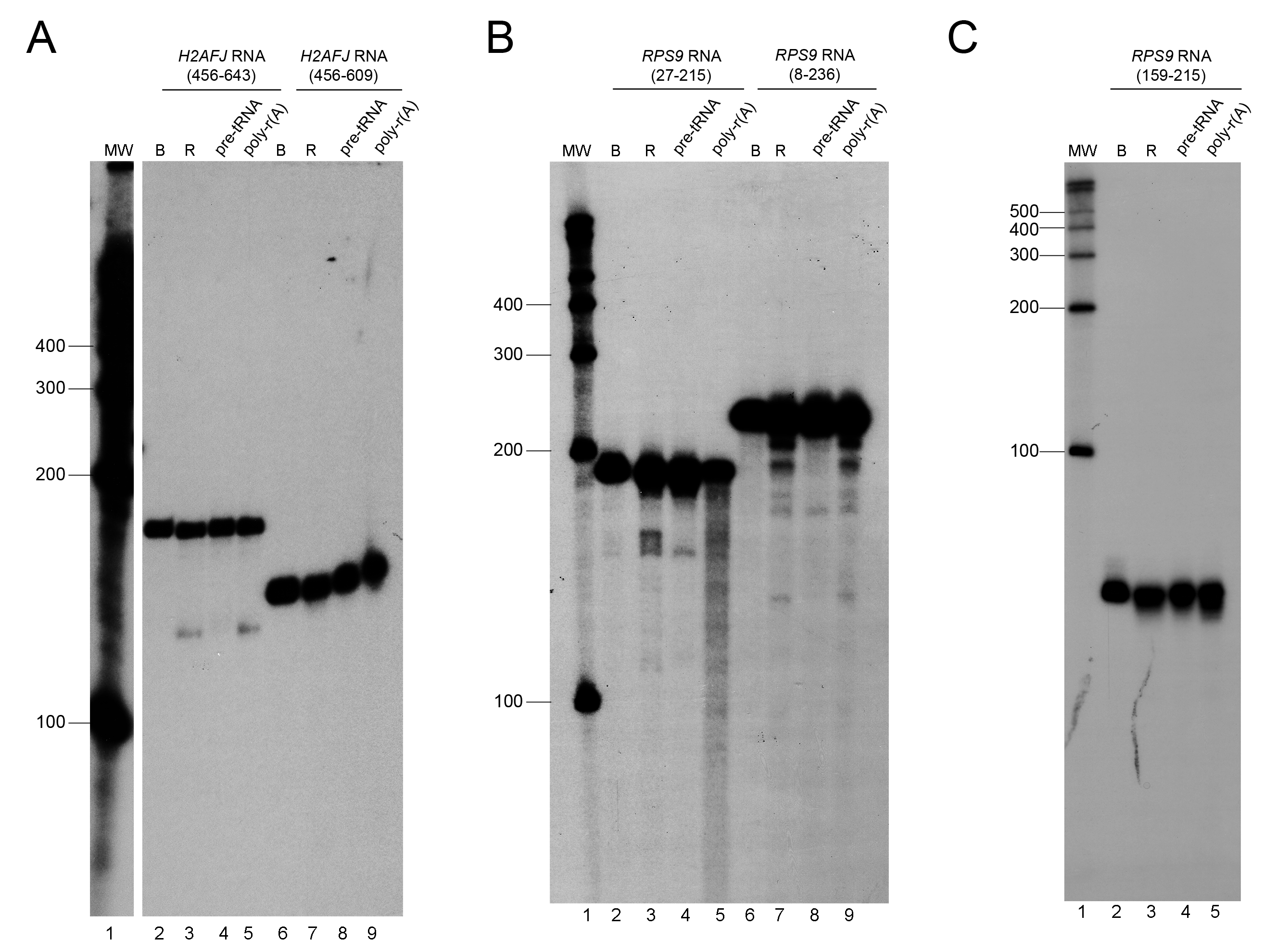

Supplement: Supplementary file 5 — Figure S5: Determination of human RNase P minimal substrate and cleavage specificity for H2AFJ and RPS9 mRNAs. Internally labelled RNAs of H2AFJ (panel A) and RPS9 (panel B and C) were incubated in reaction buffer (lanes 2 and 6), in the presence of human RNase P (lanes 3 and 7), with addition of unlabelled pre-tRNATyr (10 × molar excess) (lanes 4 and 8), or poly-r(A) (equivalent quantity by weight) (lanes 5 and 9) respectively. Lane 1 shows the century molecular markers. RPS9 (8-326) and (27-215) RNAs, incubation with human RNase P activity provided a product approximately 180 nts in length (Sup. Figure 7B) derived from a new cleavage site (not found in the full length RPS9 RNA (1-714) (Fig. 2C). This new product disappeared in the shortened RPS9 (159-215) fragment (Sup. Figure 7C), thereby probably indicating an altered structure in the shortened RPS9 transcripts (TIFF 8459 kb) [file 18_2015_1908_MOESM5_ESM.tif]

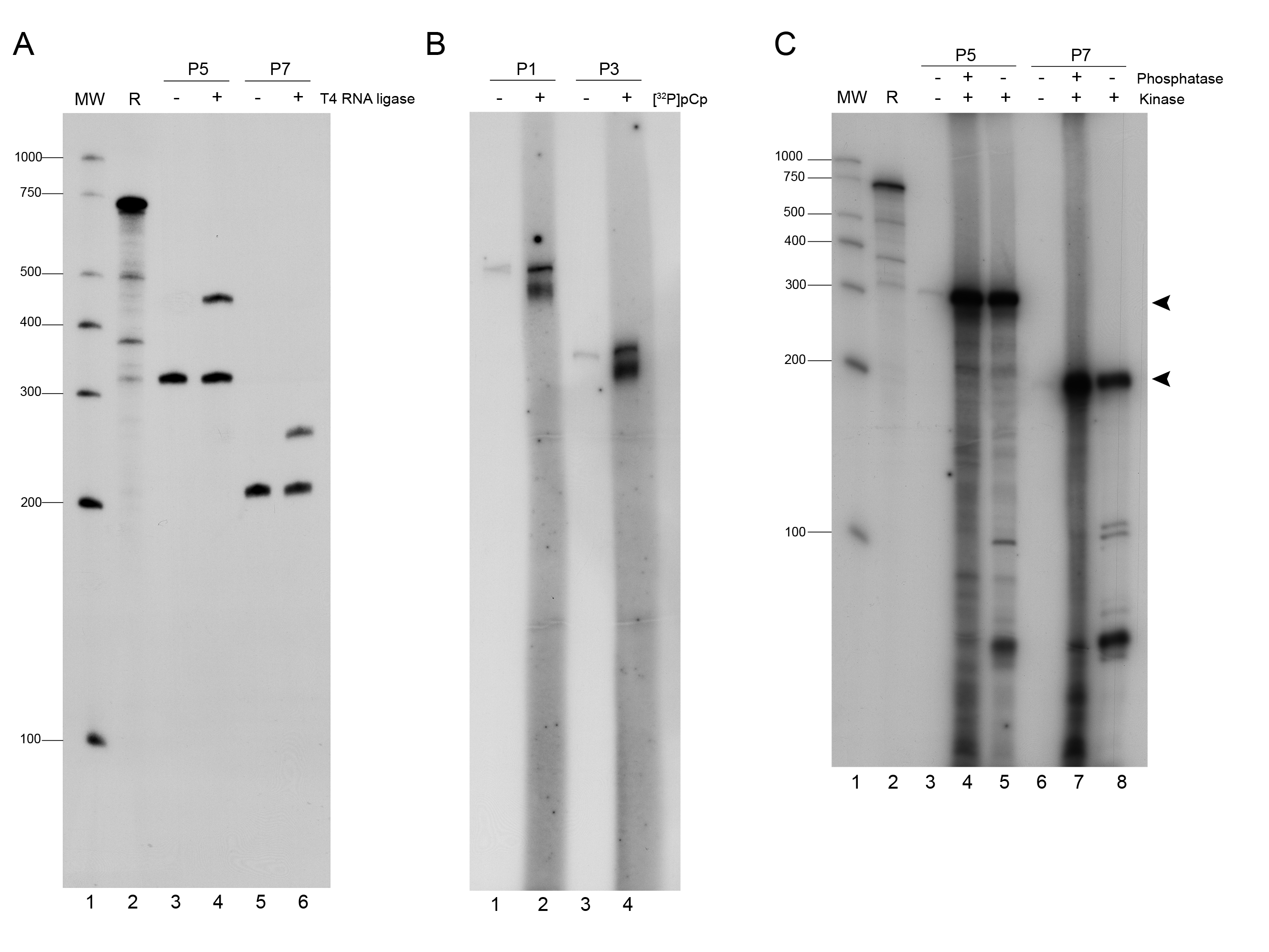

Supplement: Supplementary file 6 — Figure S6: Biochemical characterization of IFNA5 RNase P cleavage product end-groups. IFNA5 (1-700) was labelled at a sufficiently low specific radioactivity (105 dpm/µg) in order to trace its electrophoretic mobility and to permit an increase in radioactivity incorporated in the subsequent end-labelling reactions. RNase P digestion product bands P1, P3, P5 and P7 (see Fig. 2 panel A) were purified by preparative gel electrophoresis and subjected to different specific enzymatic treatments to determine the chemical groups. A) Determination of the newly generated 5′-ends by T4 RNA ligase circularization. Lane 1: molecular weight; lane 2: IFNA5 RNA subjected to a standard human RNase P reaction; lanes 3 and 5: product bands P5 and P7 incubated on ice; lanes 4 and 6: product bands P5 and P7 incubated with the enzyme. New bands in the T4 RNA ligase treatment with delayed mobility than the untreated material, corresponded to circularized P5 and P7, thus confirming their 5′-P end. B) Enzymatic determination of new 3′-end by labelling with T4 RNA ligase and [32P]pCp. Lanes 1 and 3: bands P1 and P3 incubated on ice; lanes 2 and 4: P1 and P3 in presence of the enzyme and [32P] pCp. Increase of label in the presence of enzyme and [32P]pCp is indicative of 3′-OH end in P1 and P3. C) Determination of the newly generated 5′-ends by differential phosphatase/kinase and [γ-32P]ATP treatment. Lane 1: molecular weight ladder; lane 2: IFNA5 RNA subjected to a standard human RNase P reaction; lanes 3 and 6: bands P5 and P7 incubated on ice; lanes 4 and 7: bands P5 and P7 labelled with T4 polynucleotide kinase and [γ-32P]ATP after prior incubation with phosphatase; lanes 5 and 8: treated identically as before but without alkaline phosphatase pretreatment. A characteristically low increase in label was observed in the phosphatase-treated samples, thus indicating the presence of a 5′-P (TIFF 8256 kb) [file 18_2015_1908_MOESM6_ESM.tif]

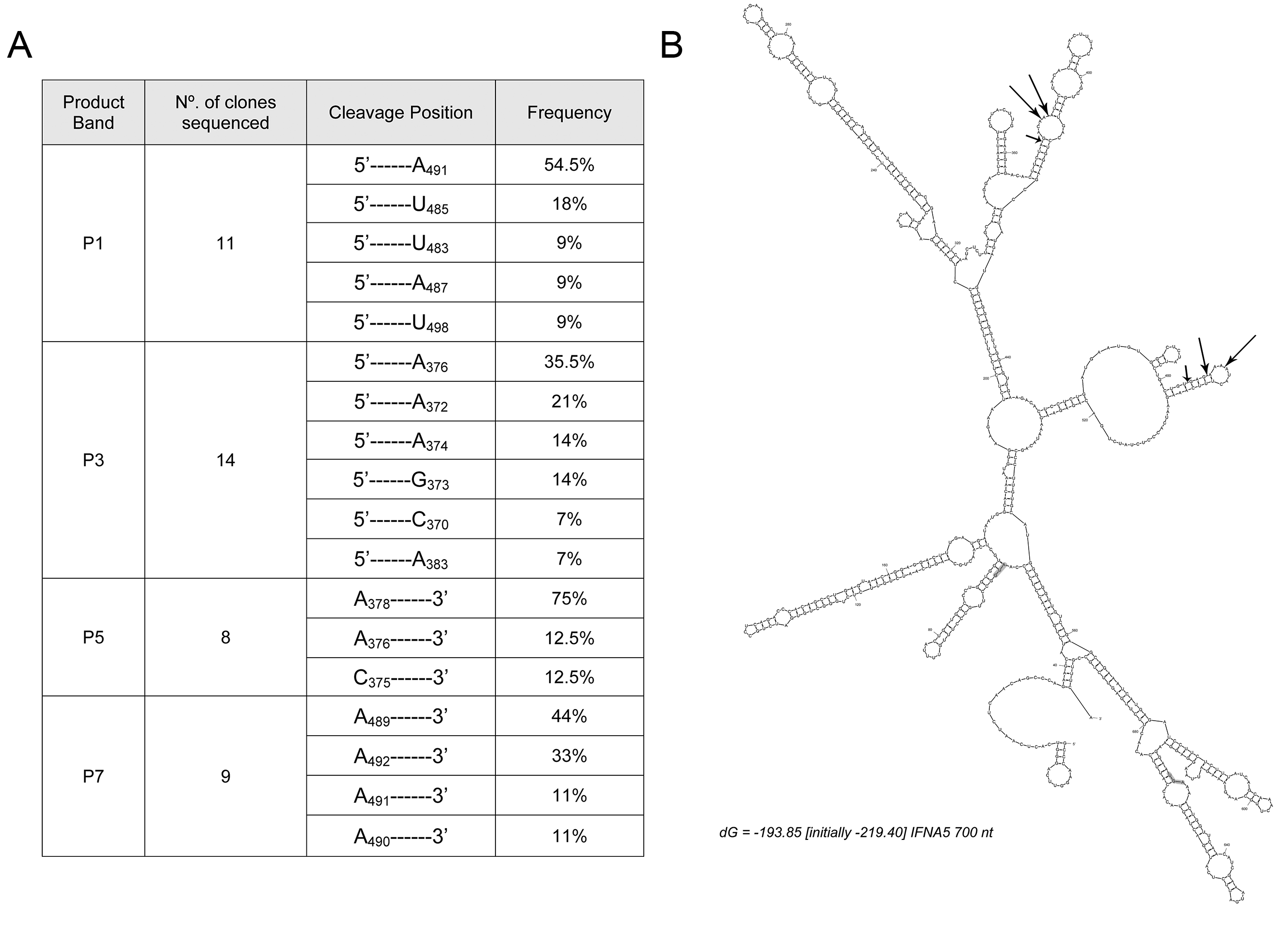

Supplement: Supplementary file 7 — Figure S7: Determination of human RNase P cleavage sites on IFNA5 mRNA by indirect sequencing. A) The table summarizes the results obtained upon subcloning and sequencing of cleavage product bands P1, P3, P5 and P7. The number of clones sequenced and the frequency of representation of clones for each cleavage position is described for each product. B) Positioning of the two most represented RNase P cleavage sites on the predicted secondary structure model for IFNA5 mRNA (using Mfold program). Arrow lengths are proportional to the observed frequency of each cleavage position (TIFF 5555 kb) [file 18_2015_1908_MOESM7_ESM.tif]

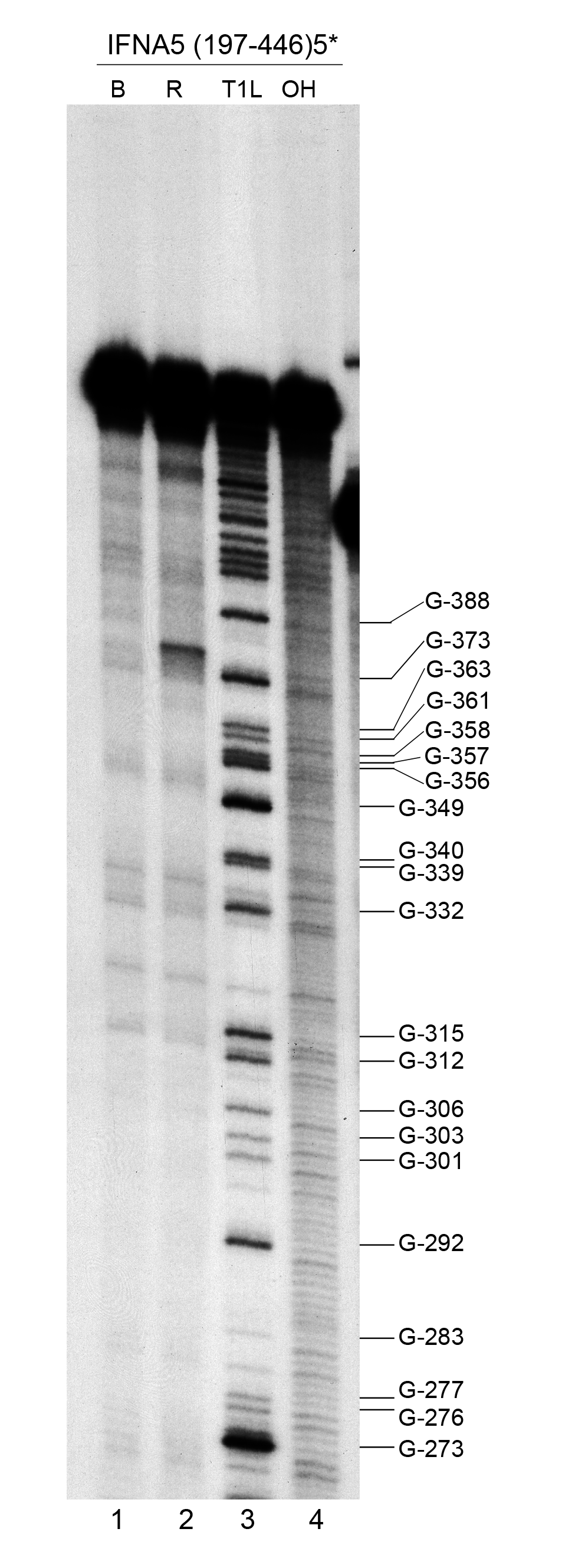

Supplement: Supplementary file 8 — Direct determination of human RNase P cleavage sites in IFNA5 by direct sequence analysis. Both transcripts were labelled at its 3′ end with [32P]pCp. IFNA5 (197–446) RNA: lane 1 RNA transcript alone incubated in buffer Arrow indicate the most prominent band (P1). Samples were electrophoresed on a 6 % denaturing polyacrylamide gels (TIFF 4082 kb) [file 18_2015_1908_MOESM8_ESM.tif]

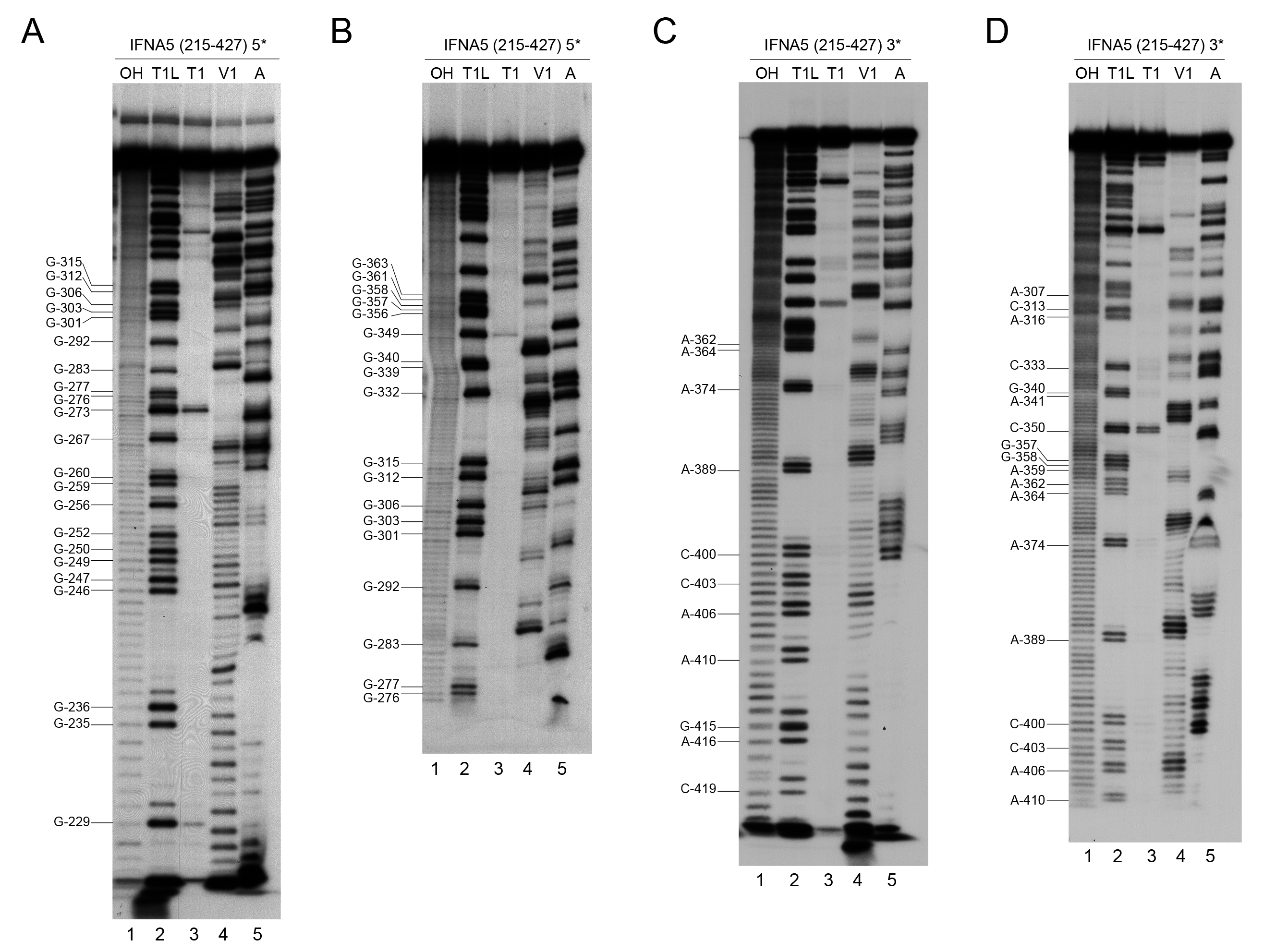

Supplement: Supplementary file 9 — Figure S9: Enzymatic probing of the secondary structure of IFNA5 RNA (215-427). Panels A and B: 5′-[32P] end-labelled RNA. Panels B and C: 3′-[32P] end-labelled RNA. For all cases: lane 1 alkaline hydrolysis reaction (OH); lane 2 RNase T1 reaction under denaturing conditions (T1L); lane 3 RNase T1 (T1), lane 4 RNase V1 (V1) and lane 5 RNase A (A) under standard conditions, respectively. Denaturing gels were at 10 % (panels A and C) or 6 % polyacrylamide (panels B and D) (TIFF 8802 kb) [file 18_2015_1908_MOESM9_ESM.tif]

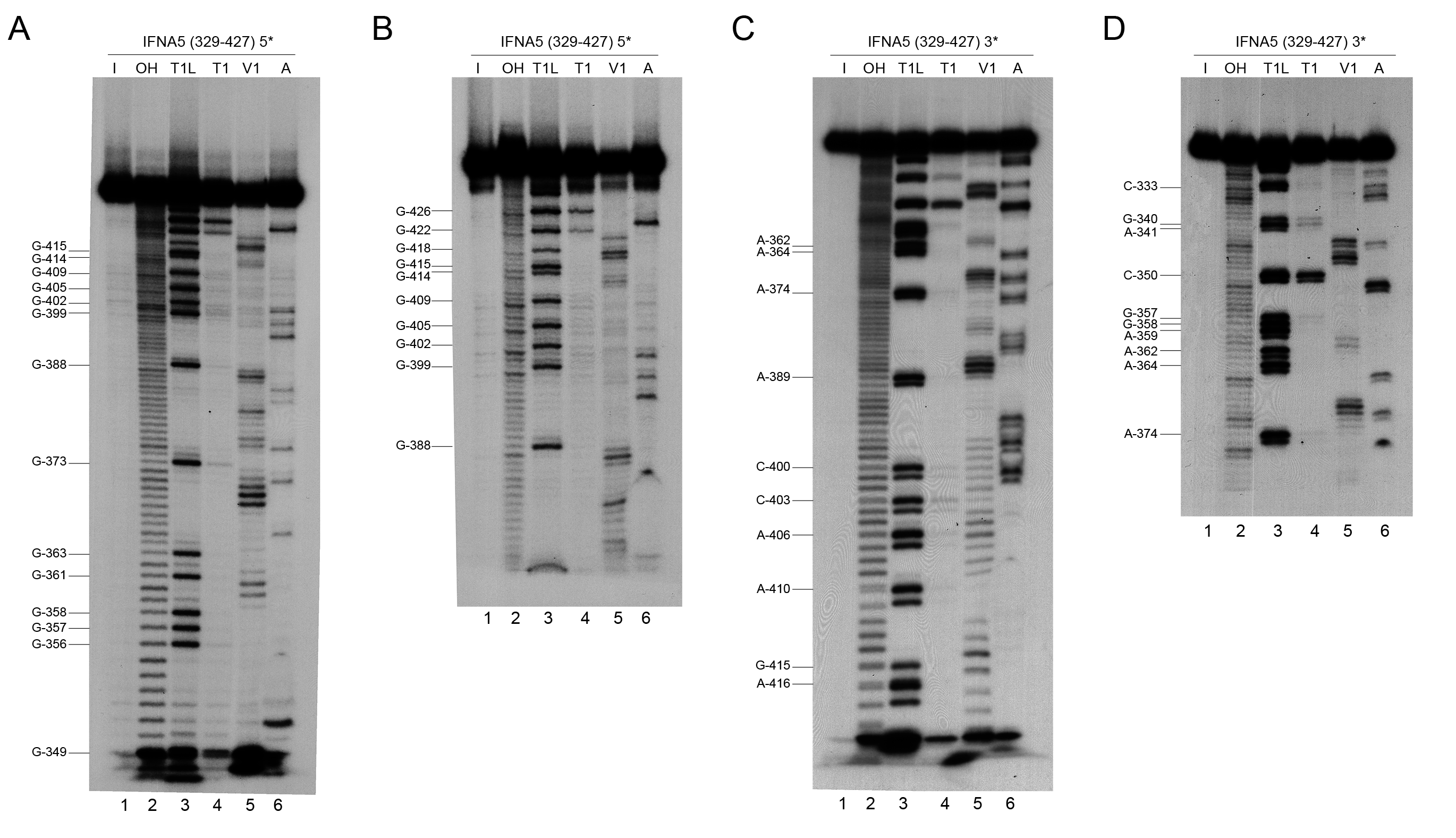

Supplement: Supplementary file 10 — Figure S10: Enzymatic probing of the secondary structure of IFNA5 (329-427). Panels A and B: 5′-[32P] end-labelled RNA. Panels B and C: 3′-[32P] end-labelled RNA. For all cases: lane 1 RNA incubated on ice (I); lane 2 alkaline hydrolysis reaction (OH); lane 3 RNase T1 reaction under denaturing conditions (T1L); lane 4 RNase T1 (T1), lane 5 RNase V1 (V1) and lane 6 RNase A (A) under standard conditions, respectively. Denaturing gels were at 10 % (panels A and C) or 6 % polyacrylamide (panels B and D) (TIFF 7101 kb) [file 18_2015_1908_MOESM10_ESM.tif]

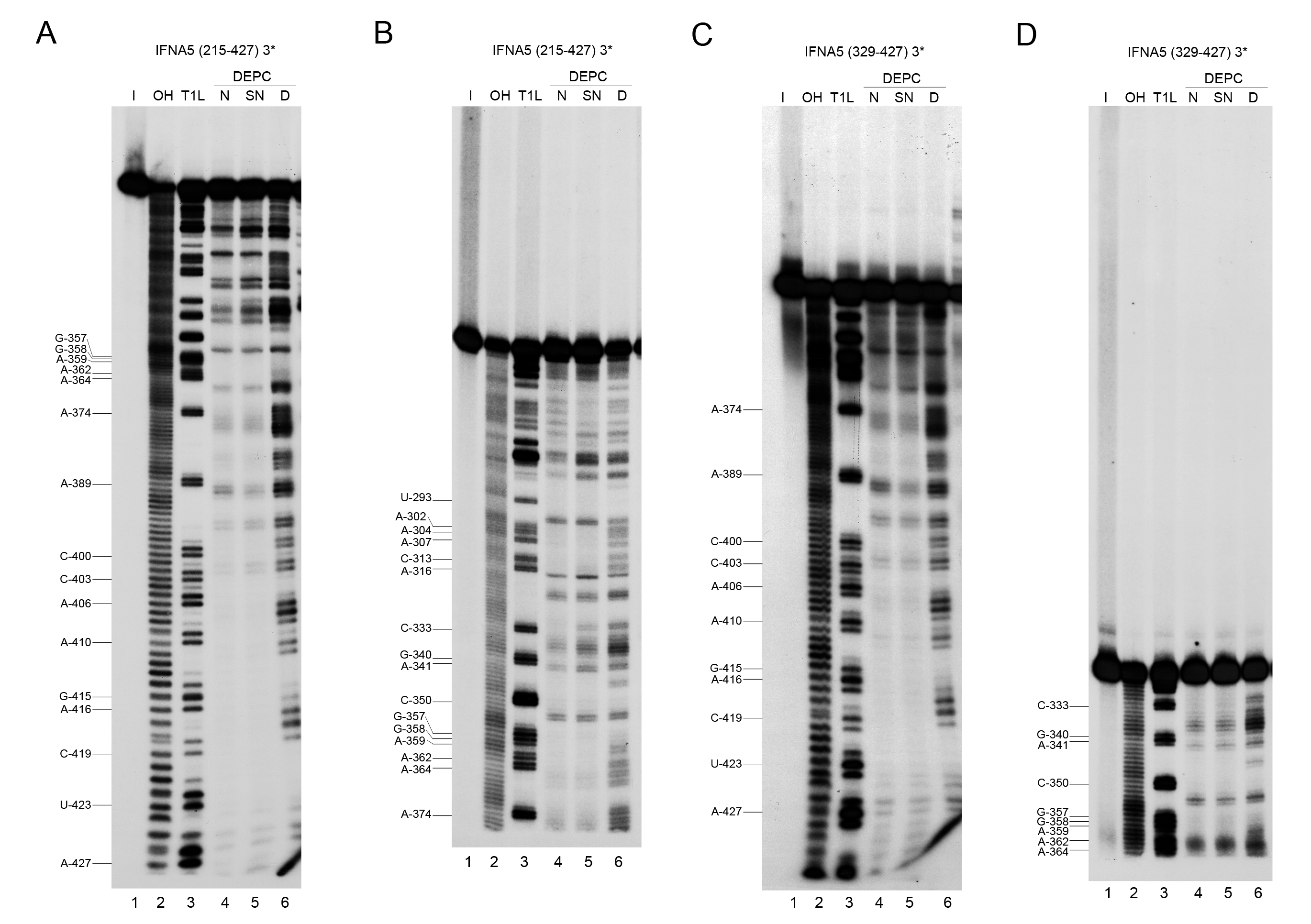

Supplement: Supplementary file 11 — Figure S11: DEPC probing of 3′ end-labelled IFNA5 RNAs (215-427) and (329-427). Panels A and B: 3′-[32P] end-labelled RNA (215-427). Panels C and D: 3′-[32P] end-labelled RNA (329-427). Lane 1 is the RNA maintained on ice (I). Lanes 2 and 3 are the products from digestion with alkali (OH) and RNase T1 under denaturing conditions (T1L), respectively. Products of aniline-treated RNAs previously modified with DEPC under native conditions (lane 4), semi-denaturing conditions (lane 5) and denaturing conditions (lane 6). Denaturing gels were at 10 % (panels A and C) or 6 % polyacrylamide (panels B and D) (TIFF 8827 kb) [file 18_2015_1908_MOESM11_ESM.tif]

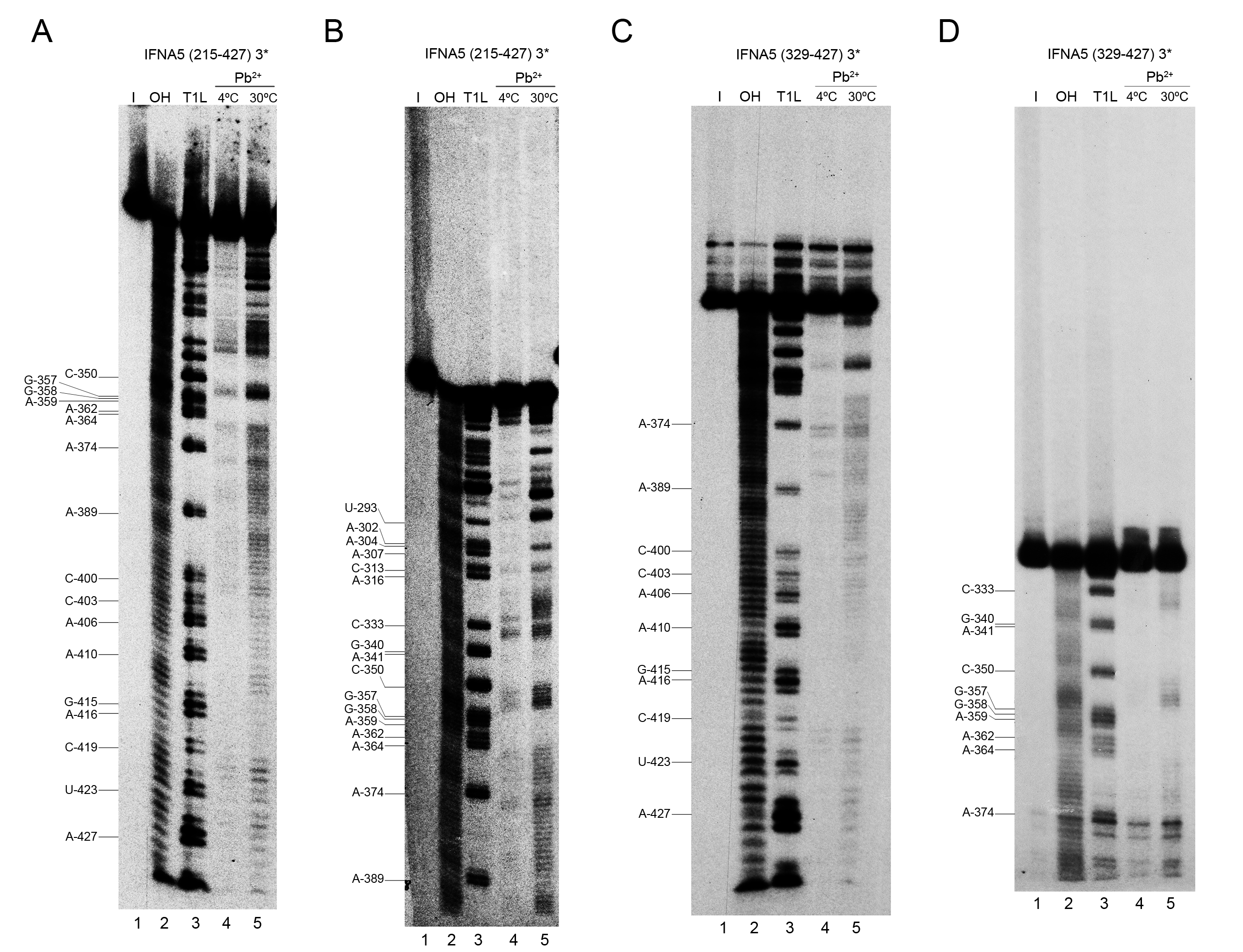

Supplement: Supplementary file 12 — Figure S2: Pb2+ probing of 3′ end-labelled IFNA5 RNAs (215-427) and (329-427): Panels A and B: 3′-[32P] end-labelled RNA (215-427). Panels C and D: 3′-[32P] end-labelled RNA (329-427). Lane 1 is the RNA maintained on ice (I). Lane 2 treatment with alkali (OH); lane 3 treatment with RNase T1 under denaturing conditions (T1L); lane 4 treatment with Pb2+ at 4ºC for 15 min and lane 5 treatment with Pb2+ at 30ºC for 20 s. Denaturing gels were at 10 % (panels A and C) or 6 % polyacrylamide (panels B and D) (TIFF 9059 kb) [file 18_2015_1908_MOESM12_ESM.tif]

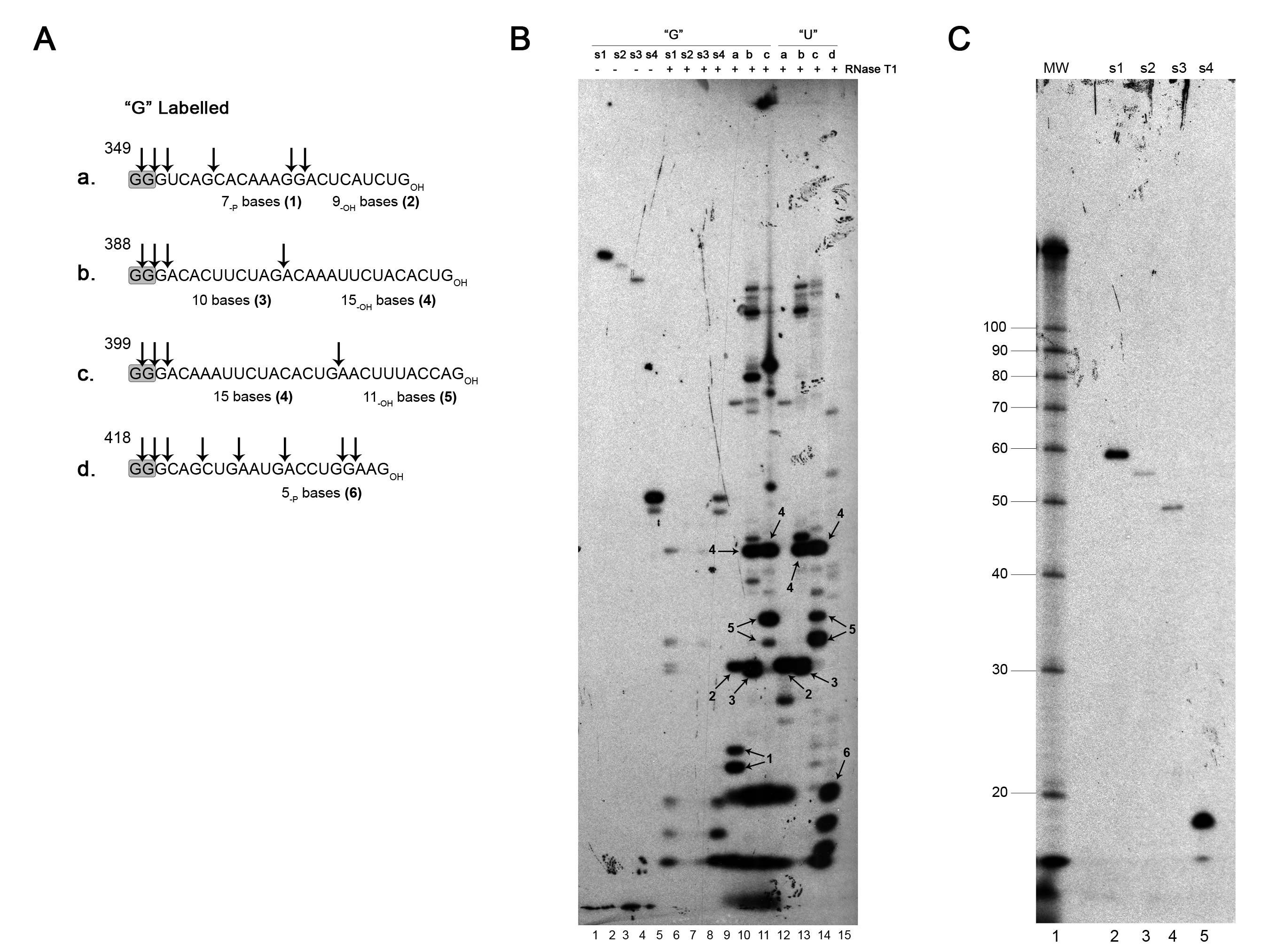

Supplement: Supplementary file 13 — Figure S13: Identification of complete RNAse T1 digestion products of IFN5A RNA (329-427) A: Sequence of the RNA markers used. 19 to 26 bases oligonucleotides sequences from RNA (329-427) employed as RNA precursors which are cleaved with RNase T1. The reaction yield a subset of short oligonucleotides (indicated by arrows, and numbered) for use as mobility controls in a high percentage denaturing polyacrylamide gel. B: The partial digestion products of [32P]-labelled IFNA5 RNA (329-427) S1 to S4 (lanes 1 to 4) were re-digested to completion with RNase T1 (lanes 5 to 8), and the products analysed in the presence of known subsets of the expected complete products (produced from the oligonucleotides described in panel A) (lanes 10-15) on 26 % denaturing polyacrylamide gel. The numbers within the gel identify the deduced band sequences described in panel A. Oligonucleotides used for mobility markers were either “G” or “U” labelled to facilitate identification. “U” labelled oligonucleotide “a” was lost during purification. C: 20 % denaturing polyacrylamide gel showing the mobility of products S1, S2, S3 and S4 (lanes 2-5) in comparison with “decade” RNA molecular weight markers (lane 1) (TIFF 8137 kb) [file 18_2015_1908_MOESM13_ESM.tif]

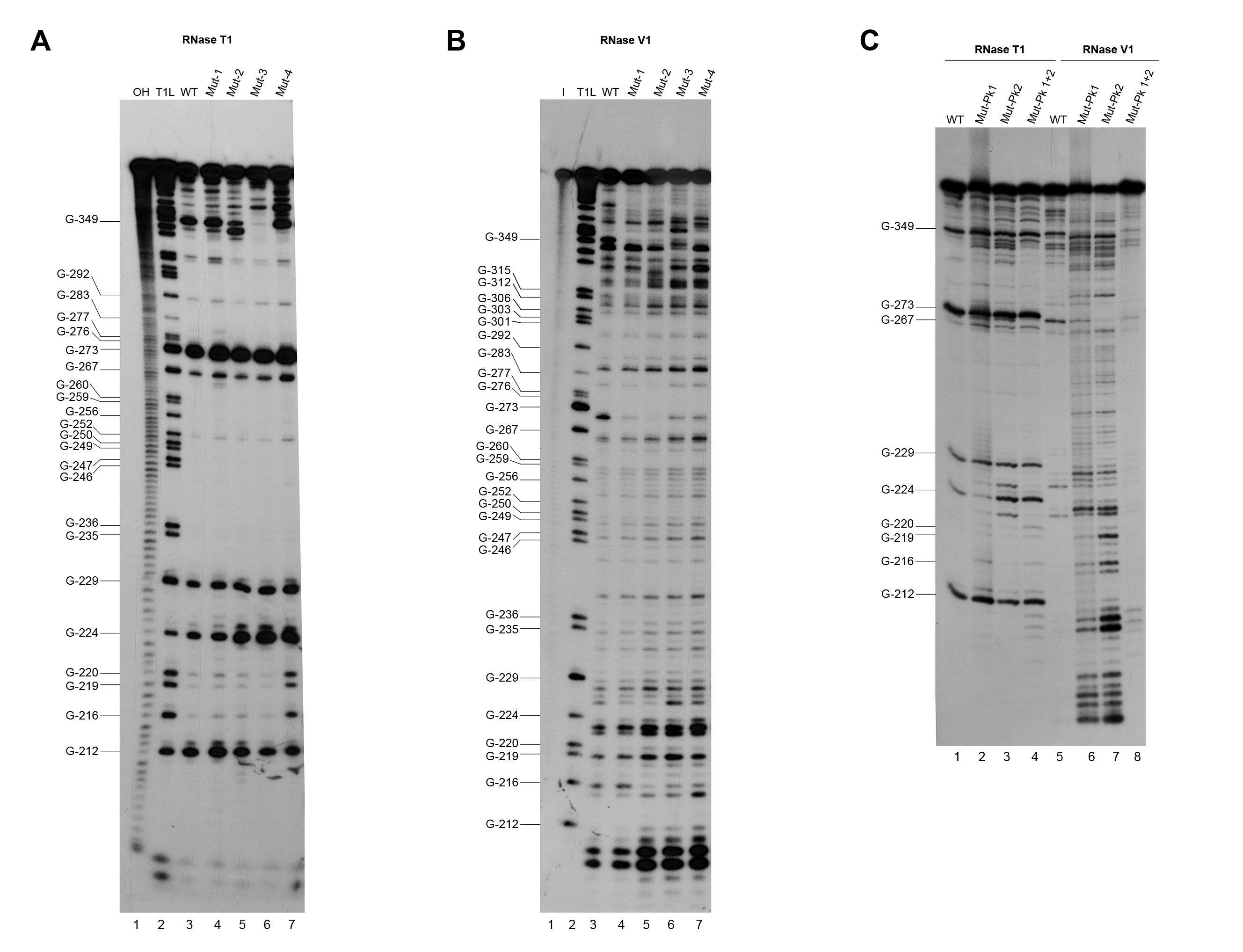

Supplement: Supplementary file 14 — Figure S14: Enzymatic probing of IFNA5 RNA (197-446) mutants. 5′-[32P] end labelled was employed for all RNA substrates. Panels A and B: Domains 1 to 4 mutants. Panel C: Pseudoknot mutants. The detailed sequences of each mutant were described in Fig. 12 of the main text. Panel A (RNase T1 reactions) and B (RNase V1 reactions): lane 1 alkaline hydrolysis reaction (OH); lane 2 RNase T1 under denaturing conditions (T1L); lanes 3 to 7 corresponded to Wt, Mut-1 to Mut-4 sequences, respectively. Panel C: lanes 1-4 (RNase T1) and lanes 5 to 8 (RNase V1) incubation of Wt sequence, Mut-Pk1, Mut-Pk2 or restoring mutant Mut-Pk1+2, respectively. Denaturing gels were at 10 % polyacrylamide. The numbers on the left indicate the point of digestion cleaved by RNase T1 under denaturing conditions (T1L), as identified with the help of the OH− sequence ladder (TIFF 4645 kb) [file 18_2015_1908_MOESM14_ESM.tif]

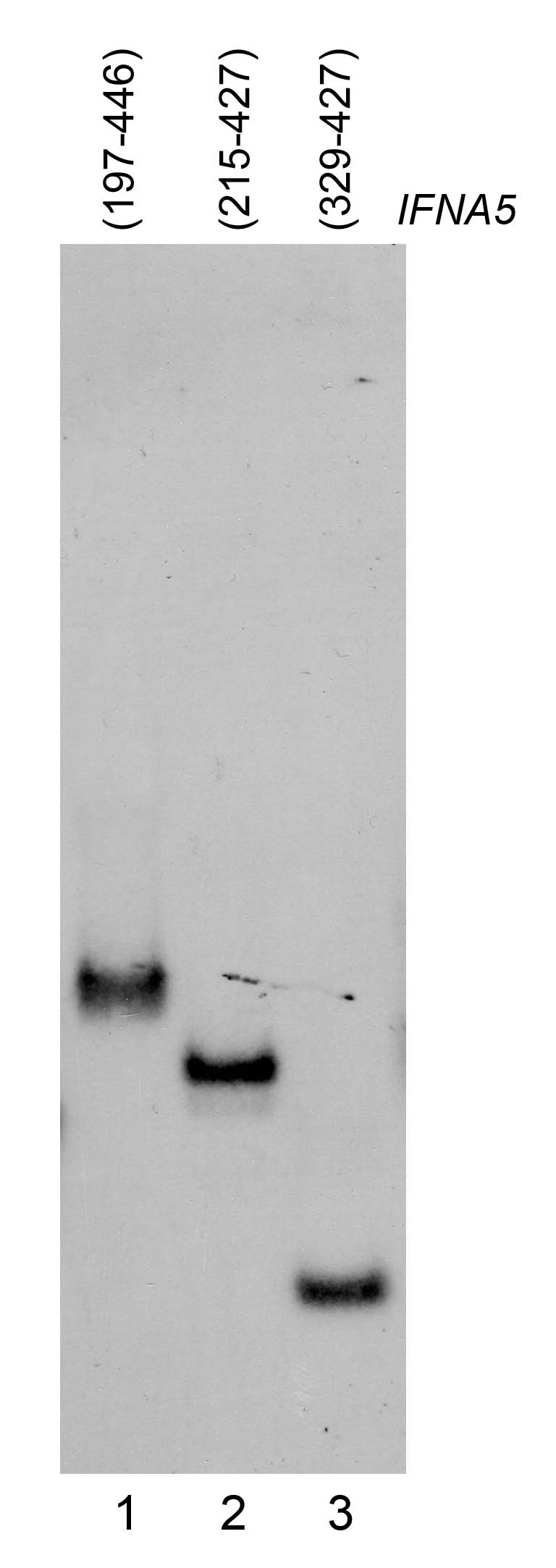

Supplement: Supplementary file 15 — Supplementary Fig. 15: Native gel of [32P] internally labelled IFNA5 RNA fragments. Autoradiography of non-denaturing 6 % polyacrylamide gel of 32P-labelled RNA samples. RNAs were denatured at 90ºC in water and left to cool in standard buffer. Each run was performed overnight at 4ºC. Lane 1: (197-446); lane 2: (215-427); lane 3: (329-427) (TIFF 1636 kb) [file 18_2015_1908_MOESM15_ESM.tif]

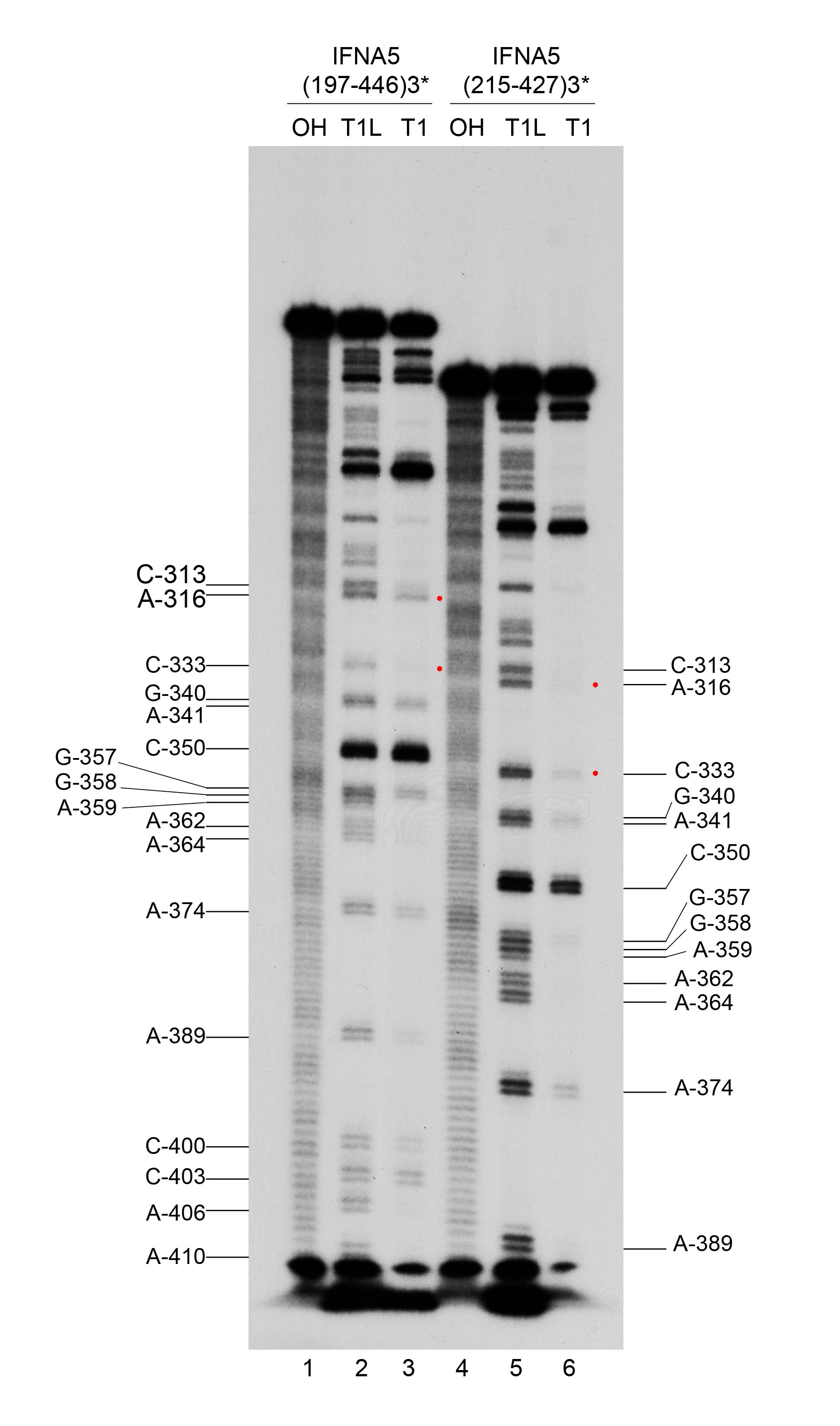

Supplement: Supplementary file 16 — Supplementary Fig. 16: Comparative RNase T1 pattern of RNAs (197-446) and (215-427). 3′ end-labelled IFNA5 (197-446) RNA (lanes 1-3) and IFNA5 (215-427) RNA (lanes 4-6) were subjected to parallel RNase T1 digestion and run in 6 % denaturing polyacrylamide gel. RNAs were treated with alkali (lanes 1 and 4), RNase T1 under denaturing conditions (lanes 2 and 5) and RNase T1 under native conditions (lanes 3 and 6). The numbers on the right indicate the point of digestion cleaved by RNase T1 under denaturing conditions (T1L), as identified with the help of the OH sequence ladders (TIFF 17839 kb) [file 18_2015_1908_MOESM16_ESM.tif]
