# Supplementary material for: Messenger RNAs bearing tRNA-like features exemplified by interferon alfa 5 mRNA
Source: Cell Mol Life Sci. 2015 Apr 22;72(19):3747–68. doi: 10.1007/s00018-015-1908-0 (PMC4565877; doi:10.1007/s00018-015-1908-0)
Supplement: Supplementary file 17 — Supplementary Table S1 (PDF 57 kb) [file 18_2015_1908_MOESM17_ESM.pdf]

Symbol	Description	*Synechocystis sp* RNase P ribozyme	GO-term biological process
TAGLN2	Transgelin	2.13	Muscle organ development
IGFBP2	Insulin-like growth factor binding protein 2	2.10	Signal transduction/cellular response to hormone stimulus/regulation of cell growth/response to stress
FTCD	Formiminotransferase cyclodeaminase	2.07	Cellular nitrogen compound metabolic process
COPZ1	Coatomer protein complex, subunit zeta 1	2.05	Vesicle-mediated transport/cellular membrane organization/intracellular protein transport
GSS	Glutathione synthetase	1.98	Response to redox state
GABARAP	GABA(A) receptor-associated protein	1.88	Protein transport/synaptic transmission/protein targeting
CHI3L1	Chitinase 3-like 1	1.88	Carbohydrate metabolic process
CRTC2	Transducer of regulated cAMP response element-binding protein	1.85	Regulation of transcription
CD99L2	CD99 antigen-like 2	1.84	Cell adhesion
SFXN4	Sideroflexin 4 variant 2	1.83	Transmembrane transport
DHRS4	Peroxisomal short-chain alcohol dehydrogenase	1.77	Oxidation-reduction process
KHDRBS1	KH domain containing RNA binding, signal transduction associated 1	1.76	Signal transduction/cell proliferation
PSAP	Prosaposin (variant Gaucher disease and variant metachromatic leukodystrophy)	1.75	Lipid metabolic process/blood coagulation
CD99L2	CD99 antigen-like 2	1.73	Cell adhesion
PC	Pyruvate carboxylase, nuclear gene encoding mitochondrial protein, transcript variant A	1.69	Carbohydrate metabolic process/glucose metabolic process/gluconeogenesis/lipid biosynthetic process
CTDSP1	CTD (carboxy-terminal domain, RNA polymerase II, polypeptide A) small phosphatase 1	1.69	Regulation of transcription from RNA polymerase II promoter/protein dephosphorylation
ROBLD3	Mitogen-activated protein-binding protein-interacting protein (MAPBPIP)	1.68	Cell growth/cellular response to amino acid stimulus/positive regulation of TOR signaling cascade
FCN2	Ficolin 2 (hucolin), transcript variant SV2	1.65	Complement activation, lectin pathway/opsonization/signal transduction
HIG2	Hypoxia-inducible protein 2	1.64	Response to stress/autocrine signaling/positive regulation of cell proliferation
HIPK2	Homeodomain interacting protein kinase 2	1.64	Apoptosis/regulation of transcription/virus-host interaction
FIS1	Tetratricopeptide repeat domain 11 (TC11)	1.63	Apoptosis/mitochondrial fission/peroxisome fission
CLPTM1L	Cisplatin resistance related protein (CRR9)	1.60	Apoptosis
GP9	Glycoprotein IX (platelet)	1.58	Blood coagulation/cell adhesion/platelet activation
PMVK	Phosphomevalonate kinase	1.58	Lipid metabolic process/protein phosphorylation
UBD	Ubiquitin D	1.58	Proteolysis/positive regulation of apoptosis
ALDH9A1	Aldehyde dehydrogenase 9 family, member A1	1.57	Cellular nitrogen compound metabolic process/oxidation-reduction process
SLC39A7	Solute carrier family 39 (zinc transporter), member 7	1.57	Transmembrane transport/ion transport
ACAD9	Acyl-Coenzyme A dehydrogenase family, member 9	1.54	Oxidation-reduction process
GANAB	Glucosidase, alpha neutral AB	1.53	Cellular protein metabolic process/carbohydrate metabolic process
CES2	Carboxylesterase 2 (intestine, liver) transcript variant 1	1.53	Catabolic process
SESN2	Sestrin 2	1.51	Cell cycle arrest
NDUFS6	NADH dehydrogenase (ubiquinone) Fe-S protein 6	1.51	Respiratory electron transport chain/mitochondrial electron transport, NADH to ubiquinone
H2AFJ	H2A histone family, member J, transcript variant 2	1.51	Nucleosome assembly
RPS9	Ribosomal protein S9	1.51	Cellular protein metabolic process/gene expression
ARPC1B	Actin related protein 2/3 complex, subunit 1B	1.51	Cellular component movement
INTS5	KIAA1698 protein	1.51	snRNA processing

Symbol	Description	***Synechocystis sp*** **RNase P ribozyme**	GO-term biological process
ADAMTS16	Disintegrin-like and metalloprotease with thrombospondin type 1 motif, 16	-1.51	Proteolysis
BMP6	Bone morphogenetic protein 6	-1.51	Multicellular organismal development
TRPC4	Transient receptor potential cation channel, subfamily C, member 4	-1.54	Transmembrane transport/ion transport
PTPN5	Protein tyrosine phosphatase, non-receptor type 5	-1.54	Protein dephosphorylation
LOXL4	Lysyl oxidase-like 4	-1.55	Oxidation-reduction process
NBN	Nijmegen breakage syndrome 1 (nibrin)	-1.63	Cell cycle/meiosis/in utero embryonic development
LHB	Lutropin subunit beta	-1.71	Cellular nitrogen compound metabolic process/signal transduction
SLC25A17	Solute carrier family 25 (mitochondrial carrier; peroxisomal membrane protein), member 17,	-2.06	Transmembrane transport/cellular lipid metabolic process
	Retrocyclin mRNA, complete cds [AF355799]	-2.08	Immune response
ADARB2	Double-stranded RNA-specific editase B2	-2.14	mRNA processing
MEIS3	Homeobox protein Meis3	-2.23	Regulation of transcription, DNA-dependent
