# Supplementary material for: Messenger RNAs bearing tRNA-like features exemplified by interferon alfa 5 mRNA
Source: Cell Mol Life Sci. 2015 Apr 22;72(19):3747–68. doi: 10.1007/s00018-015-1908-0 (PMC4565877; doi:10.1007/s00018-015-1908-0)
Supplement: Supplementary file 18 — Supplementary Table S2 (PDF 101 kb) [file 18_2015_1908_MOESM18_ESM.pdf]

	RNase T1	RNAse V1	RNases T1 and V1
Single changes	G_{216}↑ G_{219}↑ G_{220} ↑<br>G_{399}↑ G_{405} ↑ **G_{414}**↑	**G_{216}** ↓ C_{330}↑ A_{331}↑<br>C_{347}↑ **U_{343}**↓	
Helix junction	G_{212}↓↓↓<br>G_{332}↑↑<br>G_{363}↑↓↑↑	U_{343}↓↓	
Pseudoknot	G_{373} ↑<br>G_{339}↑↓$_p$$G_{340}$↓[a]<br>$G_{224}$↑↑↑	U_{315}↑ C_{419}↓<br>G_{312}↓[a]$_p$$C_{313}$↓↓↓<br>$U_{319}$↑↑$_p$$U_p$$C_{321}$↑[a]<br>$C_{318}$↑↑↑<br>$C_{226}$↓[a]$_p$$A_{227}$↓↓	
not assigned to helix junction or to pseudoknoted regions	G_{349}↓↓		G_{361}↓↑

red: $U_{206}CU_{208}$→AAA (D1), **yellow**: $U_{328}UC_{330}$→AAA (D2); **green**: $G_{358}AU_{360}$ D3, **orange:** $C_{370}UA_{372}$→AAA (D4).

D1 to D4 are domains 1 to 4.
Bases marked with [a], are nearest neighbours to bases commonly modified after different domains were mutated.
